# The Evolutionary Dynamics of a Novel Miniature Transposable Element in the Wheat Genome

**DOI:** 10.3389/fpls.2020.01173

**Published:** 2020-07-31

**Authors:** Danielle Keidar-Friedman, Inbar Bariah, Katherine Domb, Khalil Kashkush

**Affiliations:** Department of Life Sciences, Ben-Gurion University, Beer-Sheva, Israel

**Keywords:** transposable elements, *Mariam*, genome evolution, allopolyploidy, wheat

## Abstract

The discovery of *Mariam*, a wheat-unique miniature transposable element family, was reported in our previous study. We have also shown the possible impact of *Mariam* insertions on the expression of wheat genes. However, the evolutionary dynamics of *Mariam* was not studied in detail. In this study, we have assessed the insertion sites of *Mariam* family in different wheat species. *In-silico* analysis of *Mariam* insertions has allowed the discovery of two different sequence versions of *Mariam*, and that *Mariam* might have been recently active in wild emmer wheat genome (*T. turgidum* ssp *diccocoides*). In addition, the analysis of *Mariam* insertional polymorphism has facilitated the discovery of large genomic rearrangement events, such as deletions and introgressions in the wheat genome. The dynamics of *Mariam* family sheds light on the evolution of wheat.

## Introduction

Transposable elements (TEs) are mobile DNA sequences that go through transposition, i.e., change their location within the genome (Kidwell, 2002). They are divided into two classes based on their mode of transposition: Class I—RNA elements, or Retrotransposons, that transpose through a “copy and paste” mechanism *via* RNA intermediate, and Class II—DNA elements that transpose through a “cut and paste” mechanism. In most class II elements, a transposase enzyme cuts the DNA transposon at the terminal inverted repeats (TIRs), excises the TE and inserts it into the target site (Sabot et al., 2004). Transposable elements are further divided into subclasses, orders, super-families and families based on the presence of different repeats, the proteins they use for transposition and the similarity of sequences. For example, in order to be considered from the same family, TEs have to share at least 80% sequence identity in at least 80% of their coding/internal sequence or their terminal repeats (Kidwell, 2002; Wicker et al., 2007).

TEs can be found in both prokaryotes and eukaryotes, and they have been found in all eukaryote species investigated so far. Some grasses such as maize, barley and wheat possess very high amount of TEs (Kidwell, 2002; Middleton et al., 2013). While the gene content in the wheat genome is only 2%, repetitive elements represent ~85% of the genome (Charles et al., 2008; Avni et al., 2017; Clavijo et al., 2017; Appels et al., 2018). Although TEs are usually silenced due to different host epigenetic regulations as DNA methylation, RNA interference and chromatin modification, they might be activated on behalf of different stresses (Slotkin and Martienssen, 2007; Dubin et al., 2018). Abiotic stresses, biotic stresses and genomic stresses such as hybridization or polyploidization can trigger activity of TEs (Capy et al., 2000; Kashkush et al., 2003; Levy and Feldman, 2004; Lisch, 2013; Keidar-Friedman et al., 2018). Transposition can have different genetic and epigenetics effects on the host genome (Casacuberta and Santiago, 2003; Mansour, 2007; Slotkin and Martienssen, 2007; Zhao et al., 2016).

Insertions into coding regions can cause mutations that might be harmful in some cases, and in others can give rise to new protein functions and even to domestication of TEs (Rebollo et al., 2012; Zhao et al., 2016; Schrader and Schmitz, 2018; Jiang et al., 2019). Insertions into non-coding sequences might affect the expression of a gene (Kashkush et al., 2003; Li et al., 2014; Dubin et al., 2018), alternative splicing and can even lead to exonization (Lev-Maor et al., 2003; Krull et al., 2005; Schmitz and Brosius, 2011; Dubin et al., 2018; Keidar et al., 2018). Transposable elements can also be involved or induce different chromosomal rearrangements such as inversions, duplications, deletions and translocations (Gray et al., 2000; Kidwell, 2002; Bariah et al., 2020).

Wheat is one of the most important crops in the world as it provides ~20% of the total calories consumed by humans. It also has a major role in the development of agriculture as it was one of first crops that were domesticated (Appels et al., 2018; Venske et al., 2019). The origin of wheat dates back to ~5 million years ago, when three diploid ancestral species were diverged from a common progenitor. These species include the donor of A genome—*T. urartu*, the donor of B genome—a close relative of today’s *Ae. speltoides* and the donor of D genome—*Ae. tauschii*.

~ 0.5 million years ago, a hybridization event between the donors of A and B sub-genomes was followed by polyploidization and generated the tetraploid wild emmer wheat (*T. turgidum* ssp *diccocoides*, AB). ~9,000 years ago, another hybridization event between the domesticated emmer wheat (AB, *Triticum diccocon*) and the donor of D genome, was followed by polyploidization and led to the speciation of the hexaploid bread wheat (ABD) (Levy and Feldman, 2002; Feldman and Levy, 2005; Petersen et al., 2006). Wild emmer wheat grows in populations across the Middle East Fertile Crescent, in different environmental conditions. It is considered based on molecular evidence that the tetraploidization event (speciation of wild emmer) occurred in the area of Mt. Hermon and the upper Jordan valley (Israel). In Israel, there are about 20 isolated or semi-isolated populations of wild emmer that grow between Mt. Hermon in the north to Mt. Amasa in the south (Nevo and Beiles, 1989; Volis et al., 2014).

In a previous study, we have reported on the discovery of *Mariam*, a wheat-unique miniature TE-like family that was found in the coding region of a gene that encodes for 5-formyltetrahydrofolate, in two accessions of wild emmer from Mt. Hermon population (Domb et al., 2019). In this study, we have studied the dynamics of *Mariam* family in wheat species, including insertional polymorphism in various wheat species, and their association with large genomic rearrangement events. We also observed a second version of *Mariam* family, which we termed *Mariam*2. The possible role of *Mariam* during wheat evolution is discussed.

## Methods

### Genomic Data

In this study, the most updated genome drafts of five *Triticum* and *Aegilops* species were used: (1) *Triticum urartu* (TU), accession G1812 (PI428198), the progenitor of wheat A sub-genome (https://www.ncbi.nlm.nih.gov/assembly/GCA_003073215.1) (Ling et al., 2018). (2) *Aegilops tauschii* ssp. *strangulate* (AT), accession AL8/78, the progenitor of wheat D sub-genome https://www.ncbi.nlm.nih.gov/assembly/GCA_000347335.2 (Luo et al., 2017). (3) *Triticum turgidum* ssp. *diccocoides* (WE), wild emmer wheat accession *Zavitan*, genome AB (assembly version 2. WEWseq: https://wheat.pw.usda.gov/graingenes_downloads/Zavitan/) (Avni et al., 2017). (4) *Triticum aestivum* (TA), bread wheat cultivar Chinese Spring, genome ABD, IWGSC RefSeq v2.0: https://urgi.versailles.inra.fr/download/iwgsc/IWGSC_RefSeq_Assemblies/ (Appels et al., 2018). (5) *Triticum turgidum* ssp. *durum* (DW), durum wheat cultivar Svevo, genome AB, https://www.ncbi.nlm.nih.gov/bioproject/518793 (Maccaferri et al., 2019).

### Computer-Assisted Analysis of *Mariam* Insertions

Consensus sequences of the two versions; *Mariam1* and *Mariam2*, were built with Multiple Sequence Alignment (MSA) using ClustalW algorithm in Ugene software (Okonechnikov et al., 2012) according to the full-length insertions. *Mariam1* consensus sequence was built based on the 33 full-length insertions found in our previous study (Domb et al., 2019) and *Mariam2* consensus sequence was built based on the full-length insertions found using MAK - MITE analysis kit (excluding A5-2, D6-2, and D7-1 insertions that were discovered later by the following analysis). MAK (http://labs.csb.utoronto.ca/yang/MAK/) is a homology-based software, it uses a consensus sequence as query and the BLASTN algorithm with global alignment. The consensus sequences (Supp. file 1 and Supp. file 2) were used each as a query sequence in NCBI Blast+ standalone version 2.6.0 (Camacho et al., 2009). BLASTN algorithm was used with an e-value of “1e-150,” best_hit_overhang command with value “0.25” in order to avoid replications of the same sequence and specific locations of hits were extracted (commands “sstart,” “sseq”). ClustalW algorithm was used for MSA, and phylogenetic trees were constructed using Maximum likelihood with Tamura-Nei model and 100 Bootsrap replications using MegaX software (Kumar et al., 2018). Venn diagram for common insertions between species was generated using the Bioinformatic & Evolutionary Genomics tools of Ghent University (http://bioinformatics.psb.ugent.be/webtools/Venn/), based on comparison of flanking sequences of *Mariam* insertions between the different species, as explained in the following section. All insertions, their specific location and TSD can be found in **Table S1**.

### *In Silico* Analysis of Conserved *Mariam* Insertions, Their Associations With Genes and Genomic Rearrangements

Python scripts were used to retrieve *Mariam* insertions together with their flanking sequences according to their chromosomal locations, in order to examine target site duplications (TSD, in the range of 10 bp of *Mariam* flanking sequences), association with genes (in the range of 500 bp of *Mariam* flanking sequences), conservation among different wheat species (in the range of 1000 bp of *Mariam* flanking sequences) or involvement in genomic rearrangements (by analysis of ~5000 bp or more of *Mariam* flanking sequences). TSD was generated using WebLogo software (http://weblogo.berkeley.edu/logo.cgi). Each logo is composed of a stack of letters (nucleotides) for each nucleotide position in a sequence. Each letters’ height in the stack represents its relative frequency at the specific position, whereas stack width represents the relative fraction of nucleotides at that position.

In cases where the orthologous region (~1,000 bp of flanking sequences from both side of *Mariam* insertion) of an insertion site was polymorphic (presence vs. absence of a locus) among wheat species, chromosome walking analysis was performed to assesses possible genomic rearrangements events. *Mariam*-containing locus that was present in some species and absent in others was used as query in NCBI Blast+ BLASTN algorithm for chromosomal comparative analysis. If the BLASTN hits were too short or made of repetitive sequences, a larger region was used as query (by adding 10,000 bp or more to each side). In cases where no hits were found in the investigated chromosome, the whole genome of the investigated species was used as database. Following identification of the orthologues region, both regions were aligned to generate a dot plot in Ugene software v1.29.0 (Okonechnikov et al., 2012) using a minimum repeat length of 1000 bp and 90% repeat identity.

### Plant Material and DNA Isolation

Accessions of wild diploid species—*T. urartu* (TU), *Ae. searsii, Ae. speltoides* and *Ae. tauschii* (AT), as well as accessions of the polyploid species—*T. turgidum* ssp *durum* (DW) and bread wheat (*T. aestivum*, TA) were used in this study. Seeds were kindly provided by Prof. Moshe Feldman, The Weizmann institute of Science, Rehovot, Israel. A collection of wild emmer wheat (*T. turgidum* ssp. *dicoccoides*, WE) populations from five geographically isolated sites in Israel was used in this study; Mt. Hermon, Amiad, Tabgha, Jaba and Mt. Amasa. The same collection was used in previous publications (Domb et al., 2017; Domb et al., 2019). See **Table S2** for details about the plant accessions. Leaf material was harvested ~ 4 weeks post-germination. DNA was isolated using the DNeasy plant mini kit (Qiagen).

### Site-Specific PCR

Primers for a subset of insert sites were designed in Primer3 software (See primer sequences in **Table S3**, http://bioinfo.ut.ee/primer3-0.4.0/) for PCR validation. Each PCR reaction was prepared with 10 μl of PCRBIO HS Taq Mix Red (PCRBiosystems), 7 μl of ultrapure water (Biological Industries), 1 μl of the site-specific primer (10 μM) and 1 μl of template genomic DNA (~ 50 ng/μl). The PCR conditions were 95°C incubation for 2 min, 40 cycles of 95°C for 10 s, the specific annealing temperature (calculated according to each primer set) for 15 s and 72°C for 15 s. PCR products were tested on 1.5% agarose gels and visualized with ethidium bromide (Amresco). Expected product sizes were determined by a DNA size standard (100 bp ladder, SMOBIO), and for some insertions the amplified products were extracted from gel and sequenced for validation.

## Results

### Analysis of *Mariam* Insertion Sites in Wheat Genome Drafts

*Mariam* is a family of miniature transposable elements (~300 bp in length), discovered in wild emmer wheat (*T. turgidum* ssp. *dicoccoides*), lacking TIRs (terminal inverted repeats) and other characteristics of known transposable elements (Domb et al., 2019). No hits were found for any known TE from both TREP (Wicker et al., 2002) and Repbase (Bao et al., 2015). Here, we have used the consensus sequence of *Mariam* elements found in our previous study to retrieve insertions from the publicly available wheat genome drafts. Full-length (~300 bp), as well as short versions (~93 - 240 bp) of *Mariam* insertions were detected. A total of 60 insertions were found in the 5 drafts; 3 insertions were found in *T. urartu* (TU), 4 in *Ae. tauschii* (AT), 12 in durum (DW), 21 in wild emmer (WE) and 20 insertions in bread wheat (TA).

**Figure 1:**
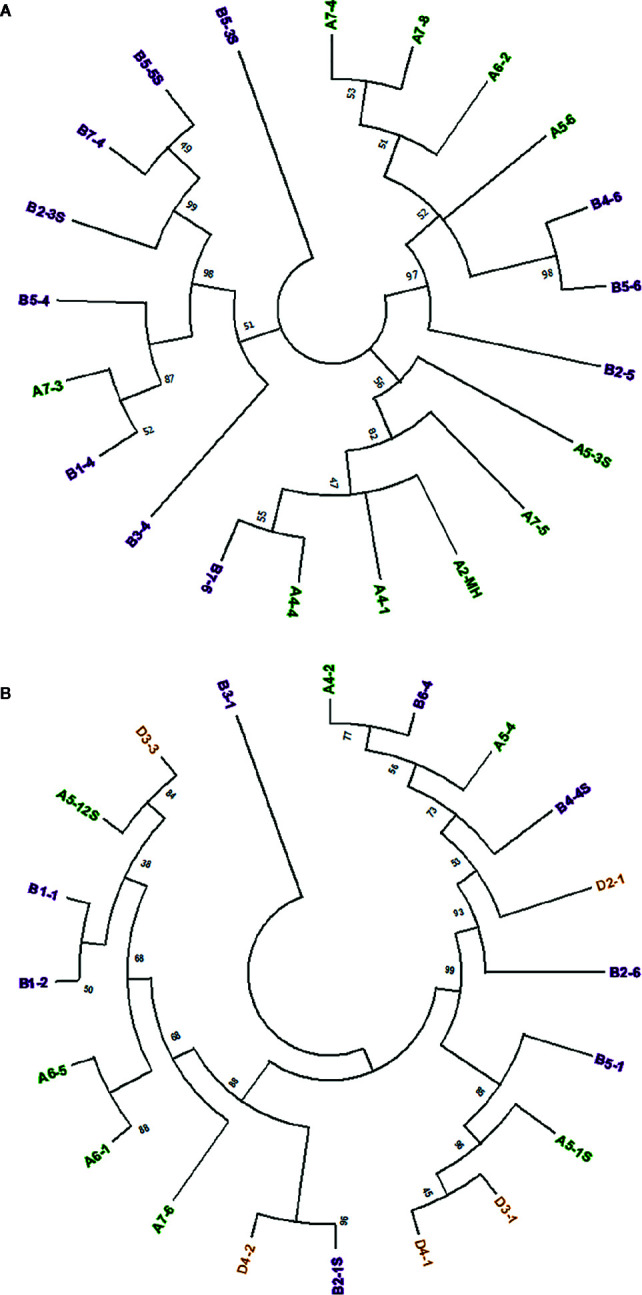
**(A)** Phylogenetic analysis of 21 *Mariam* sequences in wild emmer genome by Maximum Likelihood method. The insertions were clustered into three major groups and one insertion that was not clustered into any group (B5-3S) according to sequence similarity. **(B)** Phylogenetic analysis of 20 *Mariam* sequences in bread wheat genome by Maximum Likelihood method. The insertions were clustered into two major groups and one insertion that was not clustered into any group (B3-1) according to sequence similarity. Insertion codes are composed of the sub-genome (A/B/D), chromosome number (1-7) and a serial number. Insertions from A sub-genome are marked with green, insertions of B sub-genome are marked with purple and insertions of D sub-genome are marked with orange. A 45% cutoff parameter was used in both trees. The percentage of replicate trees in which the insertions clustered together in the bootstrap test (100 replicates) are shown next to the branches.

Sequence comparison between full-length *Mariam* insertions within A, B and D sub-genomes of wheat, and between sub-genomes might provide insights into the mobility nature of *Mariam* elements, to examine whether *Mariam* transposition is made in a genome-specific manner. To this end, a Multiple Sequence Alignment (MSA) was performed on 21 *Mariam* insertions retrieved from wild emmer genome draft. A phylogenetic tree based on the MSA clustered insertions into 3 main groups (**Figure 1A**), while one insertion (B5-3S) was not clustered into any group. The 3 cluster groups consist of insertions from both A and B sub-genomes. These clusters might indicate possible transpositions between different chromosomes (such as the case of B4-6 and B5-6 insertions) and between different sub-genomes (such as cases; B1-4 and A7-3, A4-5 and B7-6 insertions). Similar analysis was performed on the 20 retrieved *Mariam* insertions from the bread wheat genome draft (**Figure 1B**). Here, the phylogenetic tree clustered insertions into 2 main groups, while one insertion (B3-1) was not clustered into any group. Within the 2 main groups, *Mariam* insertions were clustered based on: (1) chromosome-specific manner (e.g. insertions B1-1 and B1-2, insertions A6-5 and A6-1); (2) sub-genome-specific manner (e.g. insertions D3-1 and D4-1); or (3) none specific to sub-genome or chromosome (e.g. insertions D3-3 and A5-12S, insertions A4-2 and B6-4).

**Figure 2:**
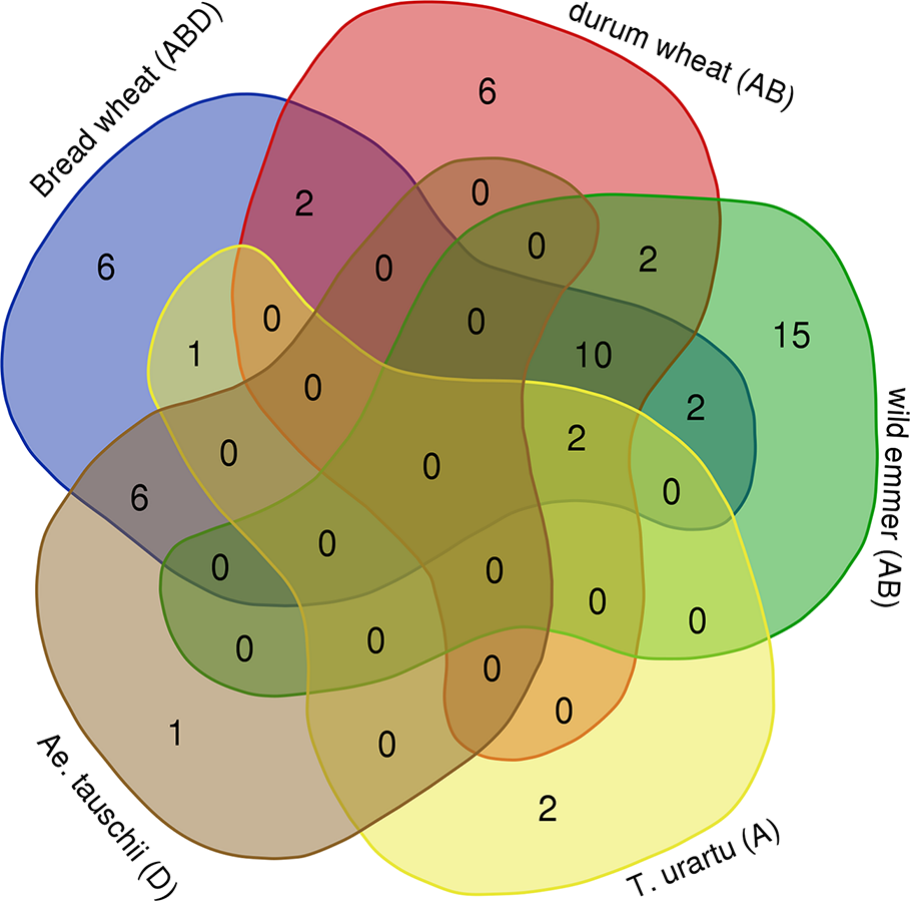
Common and unique *Mariam* insertions. Venn diagram shows the amount of common and unique insertions to each species. 2 insertions of A sub-genome were common to *T. urartu*, wild emmer, durum and bread wheat, 6 insertions of D sub-genome were common to *Ae. tauschii* and bread wheat, 10 insertions of A or B sub-genomes were common to durum, wild emmer and bread wheat. *Ae. tauschii* had only one unique insertion that was not found in bread wheat, *T. urartu* had 2 unique insertions, durum and bread wheat each had its own 6 unique insertions, while wild emmer had 15 unique insertions (not found in any other species). The diagram was generated using tools of Ghent University (http://bioinformatics.psb.ugent.be/webtools/Venn/).

In order to assess the timing of *Mariam* proliferation during wheat evolution we have performed a comparative analysis of common *Mariam* insertions in the five genome drafts of *Aegilops* and *Triticum* species. Venn diagram (**Figure 2**) shows that: (1) only two *Mariam* insertions were common to all 5 species, indicating that those are ancient *Mariam* insertions; (2) six insertions were common to both *Ae. tauschii* (D) and bread wheat (ABD), indicating those were inherited from the D donor to bread wheat ~10,000 years ago; (3) ten insertions were common to allopolyploid species- durum, wild emmer and bread wheat and absent in diploid species, indicating that those might be accompanied with allopolyploidization process; (4) six insertions were unique to durum wheat, indicating specific *Mariam* proliferation during the lifetime of durum; (5) six insertions were unique to bread wheat, indicating specific *Mariam* proliferation during the lifetime of bread wheat; and (6) 15 insertions were unique to wild emmer wheat indicating relatively higher proliferation rates of *Mariam* in wild emmer wheat.

### *Mariam2*—A Second Variant of *Mariam* Family

**Figure 3:**
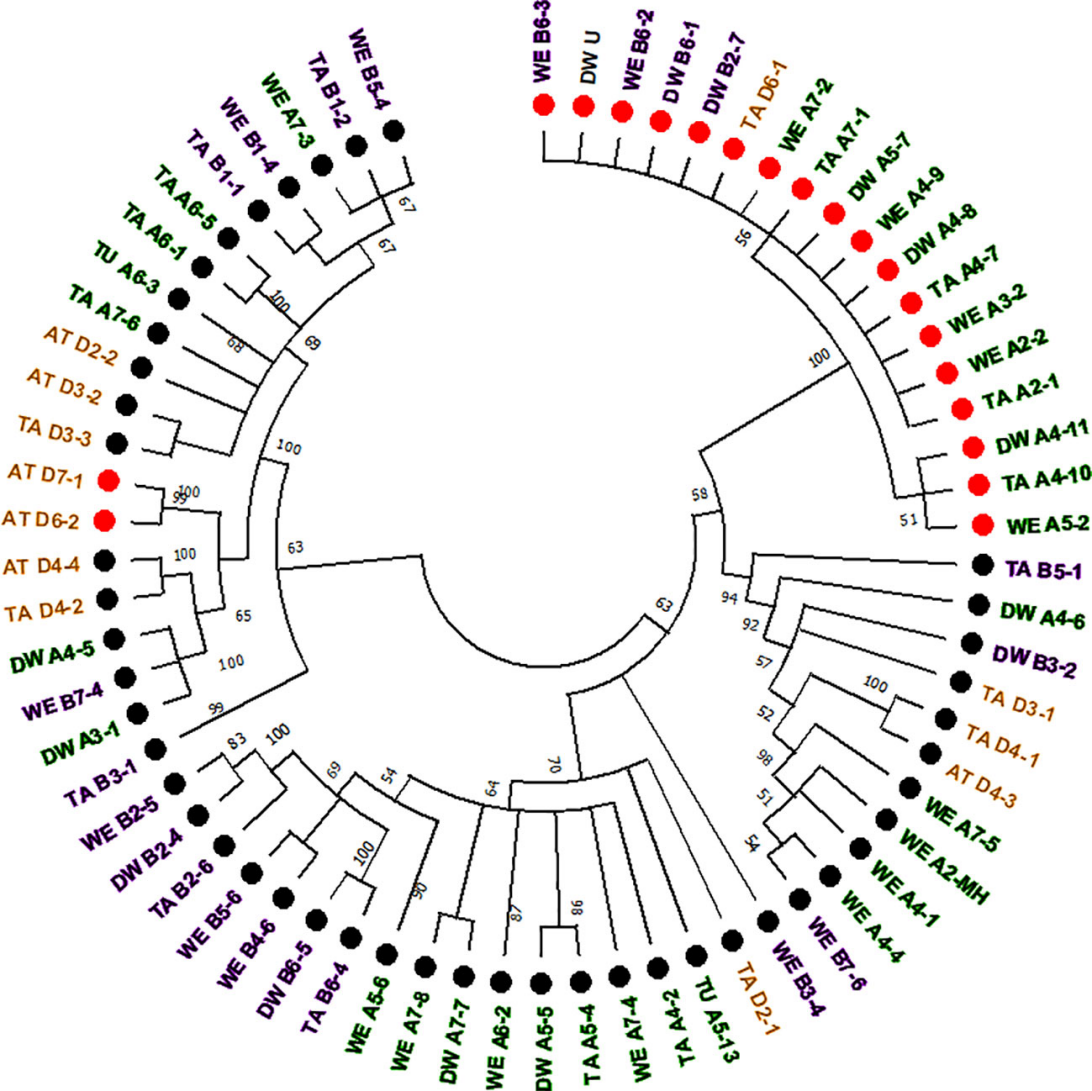
Phylogenetic tree of full-length sequences of *Mariam1* and *Mariam2*. Insertion codes are composed of the species (TA—bread wheat, AT—*Ae. tauschii*, TU—*T. urartu*, WE—wild emmer and DW—durum wheat) sub-genome (A/B/D), chromosome number (1-7\U) and a serial number. Black dots represent *Mariam1* insertions and red dots represent *Mariam2* insertions. Insertions from A sub-genome are marked with green, insertions of B sub-genome are marked with purple, insertions of D sub-genome are marked with orange and insertions of U (unknown) were marked as black. The insertions were clustered into 2 major groups, one containing mostly *Mariam1* insertions and two *Mariam2* insertions (D7-1 and D6-2). The second group was separated to smaller clusters, one containing only *Mariam2* insertions and the other clusters contained only *Mariam1* insertions. A 45% cutoff parameter was used in this tree. The percentage of replicate trees in which the insertions clustered together in the bootstrap test (100 replicates) are shown next to the branches.

**Figure 4:**
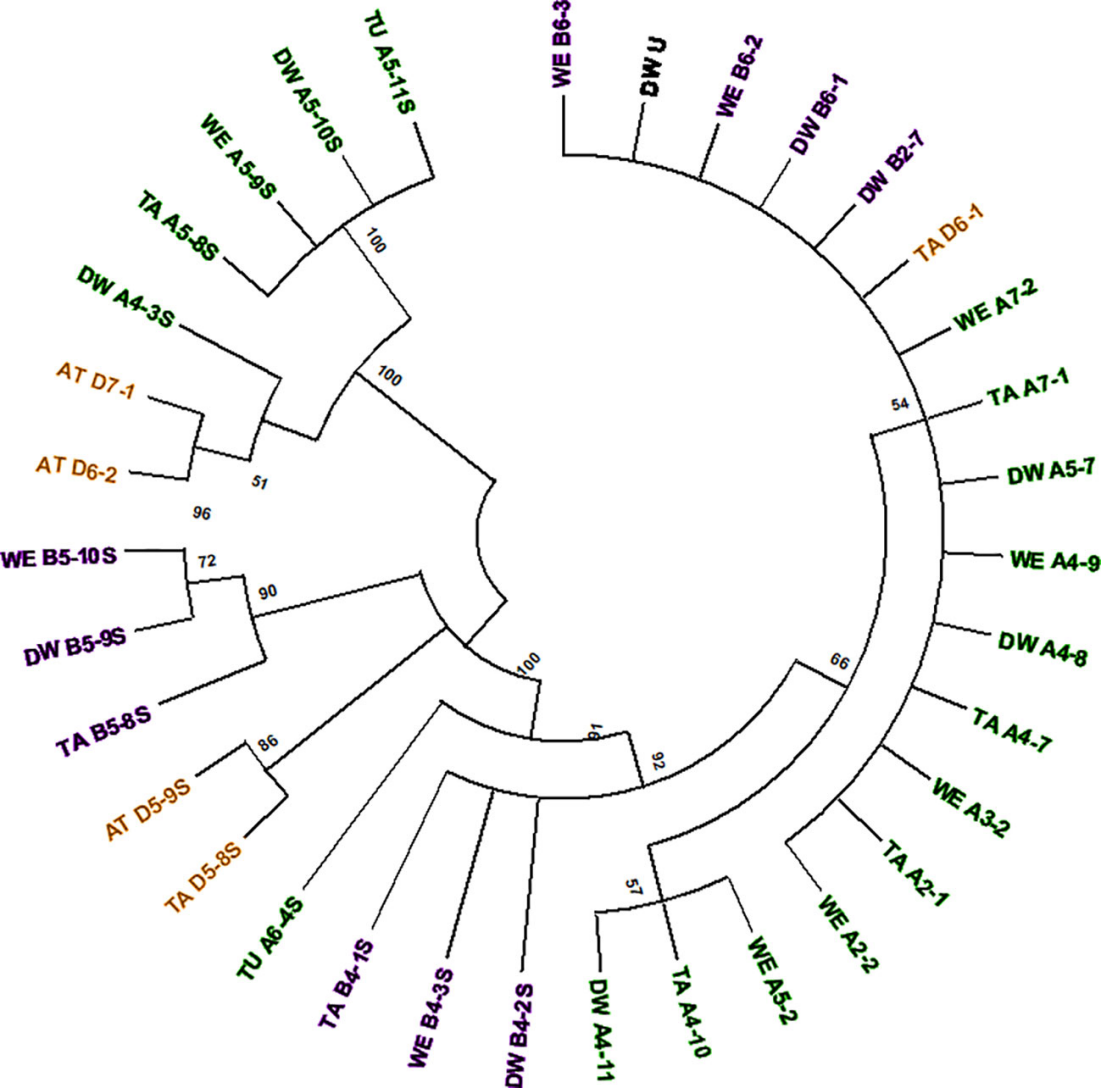
Phylogenetic tree of all *Mariam2* insertions. Insertion codes are composed of the species (TA—bread wheat, AT—*Ae. tauschii*, TU—*T. urartu*, WE—wild emmer, and DW—durum wheat) sub-genome (A/B/D), chromosome number (1-7\U) and a serial number. Insertions from A sub-genome are marked with green, insertions of B sub-genome are marked with purple and insertions of D sub-genome are marked with orange. The phylogenetic tree showed separation to two groups, while insertions were mostly clustered according to their sub-genome (A/B/D) and synthetic loci of different species. A 45% cutoff parameter was used in this tree. The percentage of replicate trees in which the insertions clustered together in the bootstrap test (100 replicates) are shown next to the branches.

The computer-assisted analysis of *Mariam* insertions led to the discovery of a second variant of *Mariam* in wheat termed *Mariam2* (291bp), suggesting this family has been divided over time into two sub-families. To validate the results and exclude sequence errors, we retrieved sequences using the second version of *Mariam* as a query sequence using MAK software and BLASTN analysis from wheat genome drafts. To this end, 34 *Mariam2* insertions were retrieved from wheat genome drafts: 10 insertions were found in wild emmer wheat, 9 in bread wheat, 10 in durum, 3 in *Ae. tauschii* and 2 in *T. urartu*. A consensus sequence of *Mariam2* was built based on MSA analysis of the retrieved sequences (**Supplementary Figure 1**). In addition, relatively high conservation (over 90% sequence similarity) among *Mariam2* full-length insertions was observed (**Supplementary Figure 2**). Analysis of target site duplication (TSD) showed that *Mariam2* full-length insertions possess a 9 bp (GTTACAAAC) TSD (**Supplementary Figure 3**). Alignment of the two consensus sequences of *Mariam1* and *Mariam*2 (using MAFFT software, **Supplementary Figure 1**) showed high sequence similarity in the first 39 bp and the last 50 bp, and two relatively large gaps; one at ~111-132 bp of the alignment suggesting deletion in *Mariam2* variant and a second gap at ~168-186 bp of the alignment suggesting deletion in *Mariam1* variant. To examine the similarity of *Mariam2* sequences to *Mariam1*, a phylogenetic tree analysis by maximum likelihood was performed for all full-length sequences. The phylogenetic tree has divided insertions into 2 major groups (**Figure 3**), the first group consists mainly of *Mariam1* insertions, and two *Mariam2* insertions (D7-1 and D6-2) that showed partial similarity to insertions of *Mariam1* (D4-2, D4-4, A4-5, A3-1 and B7-4), indicating these sequences might be intermediate between the two versions of *Mariam*. The second group was separated into sub-clusters, one consists of *Mariam2* insertions (100% bootstrap repetitions) and the other sub-clusters consist of *Mariam1* insertions. A phylogenetic tree based on Multiple sequence alignment of all *Mariam2* insertions, showed separation into 2 major groups, while insertions were clustered to smaller groups mostly by a genome-specific manner (**Figure 4**).

Analysis of common insertions of *Mariam2* in wheat species according to flanking sequences, showed one short insertion that was found within intron 1 of a gene coding for MUSE14, a TRAF domain protein (gene acc. TRIUR3_28945 and TRIDC5AG009460) and was conserved among chromosome 5 of all different wheat species and sub-genomes of wheat. Site-specific PCR validation showed a conserved insertion site in diploid and polyploid wheat accessions (**Supplementary Figure 4A**). In two other cases, the insertions found were common to the allopolyploid species (durum, wild emmer and bread wheat)—A4-7/8/9 and B4-1/2/3S (**Figure 4**). In another two cases, the insertions were common to *Ae. tauschii* and bread wheat– D5-8/9S and D6-1/2 (**Figure 4**), suggesting these are relatively old elements that were inherited from *Ae. tauschii* to bread wheat.

### Genomic Rearrangement Events of *Mariam*-Containing Sequences in Wheat

In the computer-assisted analysis we assessed events of genomic rearrangements in wheat, including large INDELs (deletions/insertions) and sequence introgressions (**Table 1**). We have assessed cases of INDELs of *Mariam*-containing sequences. For example, two *Mariam* insertions (A6-3, **Figure 3**; and A6-4S, **Figure 4**) that were found in *T. urartu* were absent in the allopolyploid genome drafts. Site-specific PCR analysis with primers flanking the insertions showed bands amplified only in *T. urartu* accessions (**Supplementary Figures 4B, C**), indicating that insertion sites of A6-3 and A6-4S underwent deletion in the allopolyploid species.

**Table 1:** *Mariam* insertions found in sequences underwent genomic rearrangements in wheat.

Insertion code	Full site^1^	Type of rearrangement
**A6-3**	TU *(T. urartu)*	Deletion probably following tetraploidization
**A6-4S**	TU *(T. urartu)*	Deletion probably following tetraploidization
**A4-3S**	DW (durum, acc. Svevo), some WE (wild emmer wheat) accessions	Introgression of a ~300 kbp sequence in durum, inversion of the ~600 kbp region flanking it
**A4-4, A4-5**	WE (wild emmer acc. Zavitan), DW (durum, acc. Svevo)	Deletion of ~440 kbp sequence and insertion of another sequence sized ~314 kbp in bread wheat
**A4-10, A4-11**	TA (bread wheat), DW (durum)	Duplication of the region including insertions A4-7 (TA) and A4-8 (DW). No duplication of the region with insertion A4-9 in wild emmer.
**B6-3**	WE (wild emmer wheat)	Deletion of a ~150 kbp sequence
**D7-1**	AT *(Ae. tauschii)*	Duplication in bread wheat.

^1^Origin of Mariam insertion.

**Figure 5:**
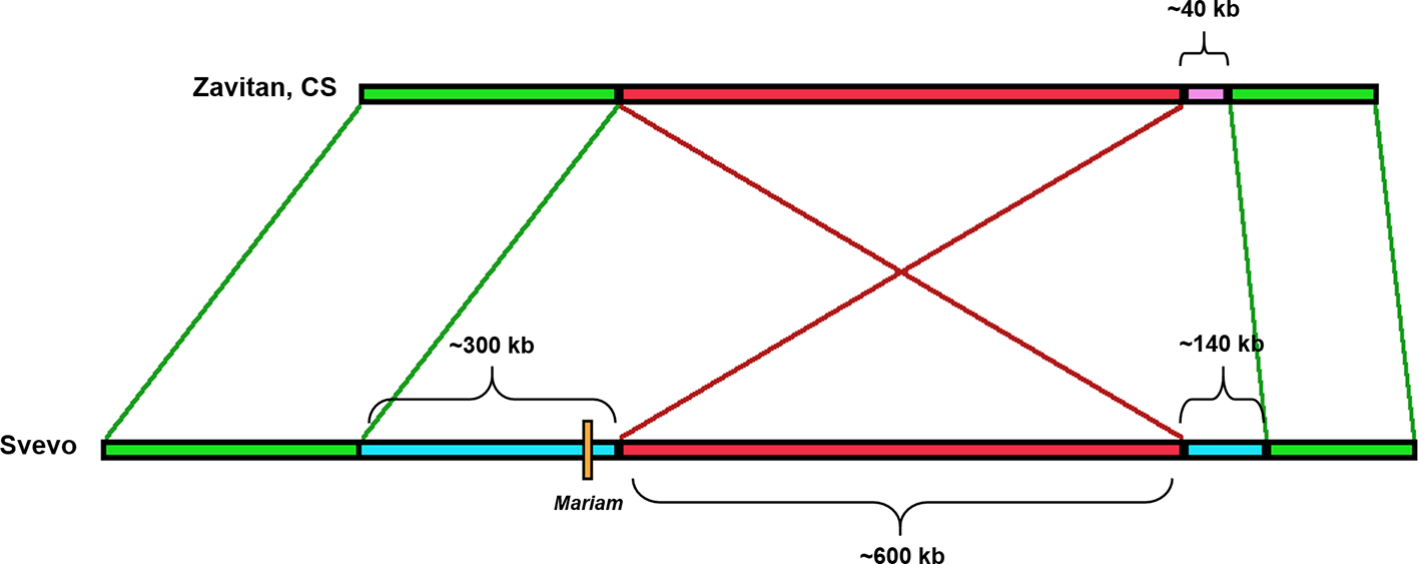
Schematic diagram of the rearrangement in which insertion A4-3S was involved in. *Mariam* (orange box) was part of a large insertion (blue box) of ~300 kbp in Svevo (DW). One side flanking this insertion showed an inversion (red box) of 600 kbp, and the other showed a sequence of ~400 kbp that was conserved in Zavitan (WE) and bread wheat (CS) as well. Upstream to the ~600 kbp inversion, an insertion of 140 kbp was found in Svevo, while a different sequence of ~40 kbp was found at the same site in Zavitan and CS. The green boxes represent conserved sites.

Insertion A4-3S (**Figure 5**) was unique to chromosome 4A in durum (coordinates 4A: 619336977-619336798) and absent in other wheat genome drafts (**Table 1**). A comparison of a larger portion of chromosome 4A of durum (Svevo) vs. chromosome 4A of wild emmer (Zavitan) and bread wheat (Chinese Spring), suggested that *Mariam* insertion is located within a region that was involved in a possible introgression event of a 289,185 bp sequence in durum (coordinates 4A: 619,072,836‬- 619,362,021)‬. Further analysis of the syntenic locus in wild emmer and bread wheat drafts showed that the downstream sequence of the introgression underwent an inversion event of ~600 kbp (coordinates in durum 4A: 619,362,021‬- 619,955,471‬), while the upstream sequence (~400 kbp) of the introgression was conserved (**Figure 5**, see also dot plot analysis in **Supplementary Figure 5A**). Upstream to the ~600 kbp inversion, an insertion of 140 kbp was found in durum (Svevo), while a different sequence of ~40 kbp was found at the same site in wild emmer (Zavitan) and bread wheat (Chinese Spring). Site specific PCR validation (**Supplementary Figure 4D**) for the flanking sequences of insertion A4-3S showed a full site in durum (Svevo), *Ae. tauschii* (acc. 603243 from Pakistan), and 3 accessions of wild emmer (TTD160-Syria, A1 and A23 (Amiad, Israel)). Note that all bands were extracted from the gel and sequenced for validation.

Molecular characterization of the locus that underwent introgression showed that this sequence includes 4 disease resistance genes (TRITD4Av1G217440, TRITD4Av1G217450, TRITD4Av1G217590, TRITD4Av1G217600) as well as Anthocyanin 5-aromatic acyltransferase (TRITD4Av1G217510) coding gene. In addition, it is part of some QTLs that are associated with biomass (QTL0782_BM-Mengistu_et_al:2016, QTL0789_BM-Mengistu_et_al:2016, QTL0788_BM-Mengistu_et_al:2016), spikelets per spike (QTL1921_4A-Roncallo_et_al._2018), height (QTL1920_4A-Roncallo_et_al._2018), spike density (QTL1062_4A-Distelfeld_et_al.) and more (see https://wheat.pw.usda.gov/jb/?data=/ggds/whe-svevo2018).

One insertion was common to durum (Svevo, A4-5) and wild emmer (Zavitan, A4-4, **Figure 1**) but was not found in bread wheat and *Ae. tauschii* (**Table 1**). Site specific PCR validation (**Supplementary Figure 4E**) with primers flanking this insertion showed full site in durum and in different accessions of wild emmer; Zavitan (Israel), TTD32 (Turkey), A16b (Amiad, Israel), J1 (Jaba, Israel), T8, T1 and T2 (Tabja, Israel). Analysis of this locus in bread wheat genome draft revealed deletion of a ~440 kbp sequence that included this insertion **(**see dot plot in **Supplementary Figure 5B** **)**. The deleted sequence contains mainly LTR retrotransposons, and CACTA transposons. In addition, a unique ~ 314 kbp insertion was found in bread wheat at the syntenic locus (coordinates: 688,952,833-689,266,972).

Insertions A4-10 and A4-11 (**Figure 4**) were found within a duplicated locus of the sequence that contains insertions A4-7 and A4-8 (**Figure 4**) in durum and bread wheat (**Table 1**). However, no duplication was detected in wild emmer, when the locus that contains A4-9 (**Figure 4**) insertion was tested.

Insertion B6-3 (**Figure 4**) was found *in-silico* only in wild emmer. Molecular characterization of the insertion site locus showed that a sequence of ~150 kbp, including the insertion in wild emmer (coordinates 6B: 499,153,784‬ - 499,306,375), was absent in durum and bread wheat genome drafts (**Table 1**, see dot plot results in **Supplementary Figure 5C**). Site-specific PCR validation using primers flanking B6-3 insertion revealed that this insertion is found in most of wild emmer wheat accessions (**Supplementary Figure 4F**) and in one accession of bread wheat (acc. 129526).

Insertion D7-1 (**Figure 4**) was mapped to chromosome 7 of *Ae. tauschii* and was not found in bread wheat genome draft. A BLASTN analysis of the regions flanking D7-1 insertion showed a ~830 bp duplication of the sequence in chromosome D7 of bread wheat (coordinates, D7:32026174-32026998 and D7: 35166235-35167066) (**Table 1**).

## Discussion

The discovery of *Mariam*, a wheat-unique miniature transposable element family, was first reported in our previous study (Domb et al., 2019). We have suggested this family can be classified into *Mutator* superfamily of MITEs (Miniature Inverted-repeat Transposable Elements) due to the 9 bp varying TSD (target site duplication) found in some insertions and the lack of TIRs (Lisch, 2002; Wicker et al., 2007). In this study, we have focused on the dynamics of *Mariam* family in different wheat species, using the genome drafts of *T. urartu* (TU, donor of A genome), *Ae. tauschii* (AT, donor of D genome), durum wheat (DW, *T. turgidum* ssp. *durum*, tetraploid of AB genome), wild emmer (WE, *T. turgidum* ssp. *diccocoides*, tetraploid AB genome) and bread wheat (TA, *T. aestivum*, hexaploidy ABD genome), as well as the genetic material of different wheat accessions.

Retrieval of *Mariam* elements from wheat genome drafts revealed different insertion lengths, ranging between 93 up to 337 bp. During the *in-silico* analysis of *Mariam* insertions, we have discovered two different variants with similar ends but a relatively different internal part, suggesting there are two sub-families of *Mariam* (**Figure 3** and **Supplementary Figure 1**). We have retrieved a total of 94 *Mariam* insertions from all 5 wheat genome drafts, of these, 60 were of sub-family *Mariam1* and 34 insertions were of sub-family *Mariam*2. A comparison of the flanking sequences of *Mariam* insertions has indicated the proportions of conserved vs. unique insertions, meaning ancient insertions inherited from progenitors or relatively new insertions indicating later activity. Wild emmer wheat presented the highest number of unique insertions suggesting that *Mariam* was probably recently active in wild emmer, similarly to our previous report on *Mariam1* based on intra-specific insertional-polymorphism analysis (Domb et al., 2019).

One of the major questions regarding the mobility of plant transposable elements is whether they transpose only within proximate regions on the same chromosome (“local hopping”) or between different chromosomes as shown in mammals (Kim, 2009; Liang et al., 2009) and even between different sub-genomes. Our analysis of *Mariam* insertions in wild emmer and bread wheat showed clusters of insertions from different chromosomes and from different sub-genomes. In some cases, clustering of insertions from homeologs chromosomes of different sub-genomes might indicate an ancient insertion in syntenic loci that has transposed in the progenitor of the diploid wheat species. In this analysis, we have found clustering of non-homeolog chromosomes from different sub-genomes, indicating a possible transposition between different chromosomes and even between different sub-genomes.

Miniature TEs are considered to be very abundant in genic regions and in some cases act as regulators of gene expression (Rebollo et al., 2012; Vaschetto, 2016; Wicker et al., 2018). Insertions into coding sequences usually disrupt gene function while insertions into introns, promotors or near genes can alter its expression or splicing (Dubin et al., 2018). Some *Mariam* insertions were found within or close to protein coding genes. For example, one insertion (A2-MH) was discussed in our previous study (Domb et al., 2019) where we showed this insertion into the CDS of a gene coding for 5-formyltetrahydrofolate cyclo-ligase leads to disruption of the ORF by insertion of a premature stop codon, that leads if translated to shorter with altered C-terminus protein. Another insertion, discussed in this study, was found within an intron 1 of a gene coding for MUSE14 and was found to be very ancient and conserved in all species and all sub-genomes suggesting that transposition occurred probably in the progenitor of diploid wheat species (over ~4 million years ago) and had a neutral or beneficial effect, as it has been fixed (domesticated) during the evolution of wheat.

Analysis of polymorphic insertions showed some cases of conserved loci with empty sites (excision/no insertion), some cases where only one side of the flanking region of *Mariam* was found and other cases in which the syntenic region was not found in other wheat species, suggesting a rearrangement has occurred at these sites (**Table 1**). We have analyzed cases of rearrangements that involved *Mariam* elements and found cases of large deletions, INDELs (deletions/insertions) and sequence introgressions in polyploid wheat species. It is important to mention that although the most updated genome drafts of *Aegilops* and *Triticum* were used, errors might occur due to incomplete sequence assembly. For this reason, wet-bench validation is required to assess the integrity of the results. In this study, we have validated using site-specific PCR analysis a subset of cases that were further analyzed (**Supplementary Figure 4**).

Transposable elements can be good evolutionary markers to study phylogenetics as well as genetic diversity among populations (Tatout et al., 1999; Queen et al., 2004; Kalendar et al., 2011; Yaakov et al., 2012; Tagimanova et al., 2015; Domb et al., 2017). Some transposable elements can be used as markers for the discovery of large-scale chromosomal rearrangements (Devos et al., 2002; Bennetzen, 2005; Kraitshtein et al., 2010; Yaakov et al., 2012; Bariah et al., 2020) and for crop improvement (Venkatesh and Nandini, 2020). Although *Mariam* is a low copy number family, its dynamics throughout the evolutionary history of wheat are quite interesting and its insertional polymorphism can be used to discover cases of large-scale rearrangements. *Mariam* was found to be a good marker for genetic diversity in populations of wild emmer wheat (Domb et al., 2019), for polymorphism in wheat species and discovery of rearrangements. With the advancement of sequencing technologies, the use of MITEs as markers in different bioinformatic analyses is becoming more and more relevant.

## Data Availability Statement

The datasets presented in this study can be found in online repositories. The names of the repository/repositories and accession number(s) can be found in the article/**Supplementary Material**.

## Author Contributions

DKF – design of the study, experiments, analyses and manuscript writing. IB –analyses of rearrangements and figures preparation, KD – design of the study and experiments, KK - design of the study and manuscript writing. All authors contributed to the article and approved the submitted version.

## Funding

This work was funded by Israel Science Foundation (grant 322/15) to KK. The authors declare that ISF is the only funding source of this work.

## Conflict of Interest

The authors declare that the research was conducted in the absence of any commercial or financial relationships that could be construed as a potential conflict of interest.
