# Supplementary material for: The Evolutionary Dynamics of a Novel Miniature Transposable Element in the Wheat Genome
Source: Front Plant Sci. 2020 Jul 31;11:1173. doi: 10.3389/fpls.2020.01173 (PMC7438880; doi:10.3389/fpls.2020.01173)
Supplement: Supplementary file 1 [file DataSheet_1.zip › New folder (2)/Data 1.DOCX]

***Mariam_1_insertions***

>Mariam1_concensus

cgaaaaggctccttcgcggccgcgcgtcgcaacgcgcgattagcctcaggcgttgtgtgc

tttcggacagggacaaacgggcccaccacatgtgtcttcaacatcaagaacaacccccgt

gatttcaactggtggggccggggtgtgccttccatattttttatctccctccgtgatttg

aattgatggggctgggatgtttccttttctatctctaatcctggcttatttaaaatggaa

agtttacttcatacctcttgtcggaaatcgctcgtcgcaacgagcgcgcgcggaatagca

tcccctc

>TA_B2-6

GTGCAACACTATCTTCAGGAGTTGCTCGAAAAGACTTATACGCGGCCGCGCGTTGCAACG

CTCAACCTGGGCATCGCGCGTACGACACTGTCGAAAAGGGCACAGGACCCACCCACATGT

CCAGACCATCCATAAAACTATGCCCCGTGATTTCAATGGTGGGGCCTAGTGTCTCCTTCC

ATATATAAAATTCTGCCCCGTGATTTCAACCGGTGGGGGGCCCGCTATGCCTGATTAATT

AGCCTCACCCTGATTTCTTACAAGGAAACTTGAGTTTCTAACGCGCTGTCGGAAATCGAT

CGTTCTCGCGCGCGCGCGTAATAGTT

>TA_B1-2

TTGAGGGATGCTATTACGCGCGCGCGCGTTAGAACGATCGATTTCCGACAGCGTGTTAGA

AACTCAAGTTTCCTTGTAAAGAAATCAGGGTGAGGCTAATTAATCAGGCATAGCGGCCCC

CCACCGGTTGAAATCACGGGGCAGAGTTTTATATATGGAAGGAGACACTAGGCCCCACTA

TTGAAATCACGGGGCAGAGTTTTATGAATGGTCTGAACATGTGGGTGGGCCCTGCGCCCT

TTCCGACAGTGTCGTACGCGCGATACCCAGATCGAGCGTTGCCACGCGCGGCCGCGTATG

AGTCTTTTCGC

>TA_B1-1

AGGGATGCTATTACGCGCGCGCGCGTTAGAACGATCGATTTTCGACAGCGTGTTAGAAAC

TCAAGTTTCCTTGTAAAGAAATCAGGGTAAGGCTAATTAATCAAGCATAGCGGGCCCCCC

AGTGGTTGAAATCACGGGGCAGAGTTTTATATATGGAAGGAGACATTAGGCCCCACTATT

GAAATCACGGGGCTTTTATGGATGGTCTGGACATGTGGGTGGGCCCTGCGCCCTTTCCGA

CAGTGTCGTACGCGCTGTGCCCAGGTCGAGCGTTGCCACGCGCGGCCGCGTATGAGTCTT

TTCGC

>TA_D2-1

GAAAAGGCTCATACGCGGCCGCGCGTTGCAACGCTCGACCTGGGCATCGCGCGTACGACA

CTGTCGGAAAGGGCGCAGGGCCCACCCACGTGTCCAGACCATCCATAAAACTCTGCCCTG

TGATTTCAATGGTGGGGCCTAGTGTCTCCTTCCATATATAAAACTCTGCCTCGTGATTTC

AACCGAAGGGGGCCTGCTATGCCTGATTAATTAGCCTCACCCTGATTTCTTTACAAGGAA

ACTTGAGTTTCTAACACGCTGTCGGAAATCCATCGTTCTAACGCACGCGCGCGTAAG

>TA_D3-1

AAAAGGCTCCTTCGCATCCGCGCATTGCGACATGCGATTAGCCTCAGGAGGTGCACGCTT

TCGGACAGGGATAATGGGGCTCACCACATGTGTCTTCAACAGAAAAAAATCACCCCCATG

ATTTCAACAGGTGGGGCTGGGGTGTGCCCTTCCATATTTTATCTCCCTCCGTGAATTCAG

TTGCTGGGGTCTGAGTGTCTCCTTTTCTATCTGTCATCCTGGCTTATTTAAAATGGAAAG

TTTACTTCGTAGGTTCTTGTCGGAAATCGCTCATCTCAATGA

>TA_A4-2

GAAAAGACTCATACGCGGCCGCGCGTGGCAACGCTCGACCTGGGTATCGCGCGTACGACA

CTGTCGGAAAGGGCGCAGGGCCCACCCACATGTCCAGACCATCCATAAAACTCTGCCCCG

TGATTTCAATAGTGGGGCCTAGTGTCTCCTTCCATATATAAAACTCTGCCCCGTGATTTC

AACCGGTGGGGGGCCCGCTATGCCTGATTAATTAGCCTCACCCTGATTTCTTTACAAGGA

AACTTGAGTTTCTAACACGCTGTCGGAAATCGATCGTTCTAACACGCGCGCGCGTAATAG

CATCCCT

>TA_B4-4S

GAAAAGACTCATACGCGGCCGCGCGTGGCAACGCTCGACCTGGGCACAGCGCGTACGACA

CTGTCAGAAAGGGCGCAGGGCCCACCCACATGTCCAGACCATCCATAAAACTCTGCCACG

TGATTTCAATAGTGGGGCCTAATATCTCCTTT

>TA_D4-2

GGATGCTATTCCGCGCGTGCTCGTTGCGACGAGCGATTTCCGACAGGAGCTACGAAATAA

ACTTTCCATTTTAAATAAGCCAGGATGAAAGATAGAAAAGGAAACACCTCGGCCCCACCA

GTTGAAATCATGGGGGAAGATAAAAGTATGGAAGGCACACCCCGGCCCCATCAGTTGTAA

TAGGGGGGGGGTTGTGATTTTTTGATGTTGAAGACACATGTGGTGGGCCCCCTTCTCCCT

GTCCGAAAGCATGCAACGCCTGAGGCTAATTGCGCGTTGCGAGGCGCGGCCGCGAAGGAG

CCTTTTCC

>TA_D4-1

AAAAGGCTTCTTCGTGGCAGCGCGTCGCAACGCGCGATTAGCTTAAGGCATTGGATGCTT

TCCTACAGGCACAAGGGGGCCCACCACATGTGTCTTCAACATCAAAAAATCACCCCCCGT

GATTTCAACCGGTGGGGCCGGGGTGCGCCTTCCATATTTTTATCTCCCCCCGTGATTTCA

ACTGGTGGGGCCGGGGTGTTTCCTTTTCTATCTCTCATCCTGGAGTATTTAAAATGGAAA

GTTTACTTCGTAGCTCCTGTCGGAAATCGCTCATCGCAACGAGCGCGCGCGGAATAGCAT

CCCT

>TA_A5-4

GAAAAGACTCATACGCGGCCGCGCGTGGCAACGCTCGACCTGGGTATCGCGCGTACGACA

CTGTCGGAAAGGGCGCAGGGCCCACCCACATGTCTAGACCATCCATAAAACTCTGCTCCG

TGATTTCAATAGTGGGGCCTAGTGTCTCCTTCCATATATAAAACTCTGCCCCGTGATTTC

AACCGGTGGGGGGCTCGCTATGCCTGATTAATTAGCCTCACCCTGAGTTCTTTACAAGGA

AACTTGAGTTTCTAACACGCTGTCGGAAATCGATCGTTCTAACGCGCGCGCGCGTAATAG

CATCCCTCT

>TA_A5-12

CTATTACGCGCGCGCGCGTTAGAACGATCGATTTCCGACAGCGTGTTAGGAACTCAAGTT

TCCTTGTAAAGAAATCAGGGTGATGCTAATTAATCAGGCATAGCGGGCCCCCCACCGGTT

GAAATCACGGGGCAGAGTTTTATATATGGAAGGAGACACTAGGCCCCACCATTGAAATTA

CAGGGCAGAGTTTTGTGGATGGTCTGGACATGTGGGTGGGCCCTGCGCCCTTTCCGAT

>TA_A5-1S

AAAAGGCTCCATTGCGGCCGCGCGTCCCGCGATTAGCCTCATGCGGTACATGCTTTCGAA

CATGGACAAAAGGGCCCAACACATGTGTCTTCAACATCAAAAAATCACCCCCACGATTTC

AACTGGTGGGGCCGAGTGTGCCCTTCCATATTTA

>TA_B5-1

AAATGGCTCCTTCGCGTCCGCGCGTCACGACGTGCGATCAGCTTCACGCCTAAGCGCTTT

GGACAGGGCAGGGGGCCCACCAGGTGTCTTTGCAACACATAAATACCCCCCCCCATGATT

TCAACTGTTGGGGCCTGGGCGTGTAATTCCATATTAATCTTCTCCCCCATGATTTATACT

GGTGGGTCTCGGGCGAGGCCGTGAATTCAAGTCTGGTGTCCACTTGTCTCTTTTTTTCTC

AGTTGCCTTGATTTAAAAAGGAAACTTTACTTCGTACCTGCCTTGTCGGAAATCGACCGC

TGCGACGCGCGCGCGGAATAGCATCACCC

>TA_A6-1

AGGGATGCTATTACGCGCGCGCGCGTTAGAACGATCGATTTCCGACAGCATGTTAGAAAC

TCAAGTTTCCTTGTAAAGAAATCAGGGTGAGGCTAATTAATCAGGCATAGCGGGCCCCCC

ACCGGTTGAAATCACGGGGCAGAGTTTTATATATGGAAGGAGACACTAGGCCCCACCATT

GAAGTCACGGGGCAGAGTTTTATGGATGGTCTGGACATGTGGGTGGGCCCTGCGGCCTTT

CCGACAGTGTCGTACGCGCGATGCCCAGGTCGAGCGTTGCAACGCGCGGCCACGTATGAG

TCTTTTCGC

>TA_A6-5

GCTATTACGCGCGCGCACGTTAGAACGATCGATTTCCGACAGCATGTTAGAAACTCAAGT

TTCCTTGTAAAGAAATCAGGGTGAGGCTAATTAATCAGCCATAGCGGGCCCCCCACCGGT

TGAAATCACGGGGCAGAGTTTTATATATAGAAGGAGACACTAGACCCCACCATTAAAGTC

ATGGGGCAGAGTTTTATGGATGGTCTGGACATGTGGGTGGGTCCTGCGGCCTTTCCGACA

GTGTCGTACACGCGATGCCCACGTCGAGCGTTGCAACGCGCGGCCGCATATGAGTCTTTT

CGC

>TA_B6-4

GAAAAGACTCATGCGCGGGCGGCCGCGCGTGGCAACGCTCGACCTGGGTATCGCGCGTAC

GACACTGTCGGAAAGGGCGCAGGGCCCACCCACATGTCCAGACCATCCATAAAACTCTGC

CCCGTGATTTCAATAGTGGGGCCTAGTGTCTCCTTCCATATATAAAACTCTGCCCCGTGA

TTTCAACCGGTGGGGGGCCCGCTATGCCTGATTAATTAGCCTCACCCTGATTTCTTTACA

AGGAAACTCACGCTGTCGGAAATCGATCGTTCTAACGCGCGCGCGTAATAGCAT

>TA_A7-6

CTCCCTCCATTCCAAAATATAGTGCGCCCGCGCTTCCCGAGGTCCAACTTTGACCATAAA

TTTAACCAACGAGACCAACTGCGGCGGAAGAAAAAATTATATAATTGAAAACTTCTTTCG

AATACGAATTCACTGATATAATTTTTGCTCCCGCCGCAATCGGTCTTGGTAGTTAAATTT

ACGGTCAAAGTTGAAGCACGTGGATAGAGGAAGCACTACATTGTGGAATGGAGGGAGTAT

ATGGAAGGAGACACTAGGCCCCACTATTGAAATCACGGGGCAGAGTTTTATGGATGGTCT

GGACATGTGGGTGGGCCCTGCGCCCTTTCCGACAGTGTCGTACGCGCGATACCCAGGTCG

AGCGTTGCCACGTGCGGCCGCGTATGAGTCTTTTCGT

>TA_B3-1

AAAAGTTAAAAAAGAGAGCGCCAAATTACTAAGTTCATCAAAAAAGAGAAGAGGGCTAAA

CTCCTTGTAGCTTCGCTTTGTGAGACGTGCGTCGACCAATACAAAATATCAGATTCATAC

GTGTCAAATGTAGTAGAAAGTGACAGTAAACATTATTTGAGTTTTGATAAAGCTAGCCAT

GATGGCTTTCCCCAAAAAAGAAGTAGAAAGTGACAGTATTAGCCGCGATCCTTTAGCCCA

TACGGCTGGTTCTGCCTGGTATCTTCTTTTCATTTGCAACGGATGGCTTTGAATTTGGAC

TG

>TA_D3-3

ATGCTATTACCCGCGCGCGCGTTAGAACGATCGATTTCCGACAGCGTGTTAGGAACTCAA

GTTTCCTTGTAAAGAAATCAGGGTGATGCTAATTAATCAGGCATAGCGGACCCCCCACTG

GTTAAAATCACGGGGCAGAGTTTTATATATGGAAGGAGACACTAGGCCCCACCATTGAAA

TCACGGTCAGAATTTTGTGGATGGTCTGGACATGTGGGTGGACCCTGCGCCCTTTCCGAC

AGTGTCGTACGCGCGATGCCCAGGCCGGGCGTTGCAACGCGCGGCCGCGTGTGAGCCTTT

TCGCCA

>TA_B2-1

TAAGGCGGGATGAGAGATAGAAAAGGAAACATCCCAGCCCCACCAGTTGAAATCACGGGG

GGACATAAAAATATGAAAGGCACACCCCGACCCCACCAGTTGAAATCACGGGGGTTTTTT

GATATTGAAGACACATGCGGTGGGCCCCTTTGTCCCTGTCCGAAAGCATGCAACGCCTGA

AGCTAATCCCGCGTTGCGACGCGCGGCCGCGAAGAAGCCTTTTCG

>DW_B2-4

GAAAAGACTTATACGCGGCCGCGCGTTGCAACGCTCAACCTGGGCATCGCGCGTACGACA

CTGTCGGAAAGGGCACAGGACCCACCCACATGTCCAGACCATCCATAAAACTATGCCCCG

TGATTTCAATGGTGGGGCCTAGTGTCTCCTTCCATATATAAAACTCTGCCCCGTGATTTC

AACCGGTGGGGGGCCCGCTATGCCTGATTAATTAGCCTCACCCTGATTTCTTACAAGGAA

ACTTGAGTTTCTAACGCGCTGTCGGAAATCGATCGTTCTCGCACGCGCGCGTAATAGT

>DW_B2-2

TAAGGCGGGATGAGAGATAGAAAAGGAAACATCCCAGCCCCACCAGTTGAAATCACGGGG

GGACATAAAAATATGAAAGGCACACCCCGACCCCACCAGTTGAAATCACGGGGGTTTTTT

GATATTGAAGACACATGCGGTGGGCCCCTTTGTCCCTGTCCGAAAGCATGCAACGCCTGA

AGCTAATCCCGCGTTGCGACGCGCGGCCGCGAAGAAGCCTTTTCG

>DW_B3-2

GGAAAAGGCTCCTTCATGGCCGCGTGTTGCAACGCACGATTAGCCTCAGCTGTGCATGCT

TACGAATAGGTACAAGGGGCCCACCACATGTGTCTTGAACATAAAAACTCACCCCCCGTG

ATTTCAACTGGTGGGGTCGGGGTGTGCCCTTCCATATTTTTATCTCCCACCGTGATTTCA

GCTATTGGGGTCCGGTGTATCCTTTTTTATTTCTCATCGTTGCGCATTTAAAATGGAAAG

TTTACTTCATAATCTCTTGTCGGAAATAGCTTGTCGCAACGAGCGTGCGTGGAATAGCAT

CCCT

>DW_A3-1

GATGCTATTCCGCGCGCGCTCGTTGCGACGAGCGATTTCCGACAAGAGGTATGAAGTAAA

CTTTCCATTTTAAATAAGCCAGGATTAGAGATAGAAAAGGAAACATCCCAGCCCCATCAG

TTCAAATCACGGAGGGAGATAAAATATATGGAAGGCACACCCCGGCCCCACCAGTCGAAA

TCATGGGGGTTGATTTTCTTGATGTTGAAGACACATGTGGTGGGCCCGTTTGTCCTTGTC

CGAAAGCACACAACGCCTGAGGCTAATCGCGCGTTGCGACGCGCGGCCGCGAAGGAGCCT

TTTCGT

>DW_A4-5

ATGCTATTCCGCGCGCGCTCGTTGCGACGAGCGATTTCCGACAAGAGGTATGAAGTAAAC

TTTCCATTTTAAATAAGCCAGGATTAGAGATAGAAAAGGAAACATCCCAGCCCCATCAGT

TCAAAACACGGAGGGAGATAAAATATATGGAAGGCACACCCCGGCCCCACCAGTTGAAAT

CACGGGGGTTGATTTTCTTGATGTTGAAGACACATGTGGTGGGCCCGTTTGTCCCTGTCC

GAAAGCACACAACGCCTGAGGCTAATCGCGCGTTGCGACGCGCGGCCGCGAAGGAGCCTT

TTCGC

>DW_A4-6

AAAAGGCTCCTTCACAGCCGCGCGTCGCAACGCGCGATTAGCCTCACGCACTGCACGCTT

TTAGACAGGGGTACATAGGGCCCCACCACATGAGTGTGCAACAAAAACCCCTCCCCCTCC

CCGTGAATTCAACTGGTGGGGCCGGGGCGTGCCCTTCCATATTTTATCCCCCTCCCCCCA

CACCCCTTGAATTCAGTTGGTGGGGGTGGGACTTATCCATTTTTATCTCTCACCCTAGCT

TATTTAAAAGGGAAGGTTTACTTCCTAAGCTCTTGTCGAAACTGCTCGTCTCGATGCACG

TGCACGGAATAGAATT

>DW_B4-5S

AGGAGATATTAGGCCCCACTATTGAAATCACGTGGCAGAGTTTTATGGATGGTCTGGACA

TGTGGGTGGGCCCTGCGCCCTTTCTGACAGTGTCGTACGCGCTGTGCCCAGGTCGAGCGT

TGCCACGCGCGGCCGCGTATGAGTCTTTTCGT

>DW_A5-5

GAAAAGACTCATACGCGGCCGCGCGTGGCAACGCTCGACCTGGGTATCGCGCGTACGACA

CTGTCGGAAAGGGCGCAGGGCCCACCCACATGTCTAGACCATCCATAAAACTCTGCTCCG

TGATTTCAATAGTGGGGCCTAGTGTCTCCTTCCATATATAAAACTCTGCCCCGTGATTTC

AACCGGTGGGGGGCTCGCTATGCCTGATTAATTAGCCTCACCCTGAGTTCTTTACAAGGA

AACTTGAGTTTCTAACACGCTGTCGGAAATCGATCGTTCTAACGCGCGCGCGCGTAATAG

CATCCCTCT

>DW_A5-2S

AAAAGGCTCCATTGCGGCCGCGCGTCCCGCGATTAGCCTCATGCGGTACATGCTTTCGAA

CATGGACAAAAGGGCCCAACACATGTGTCTTCAACATCAAAAAATCACCCCCACGATTTC

AACTGGTAGGGCCGAGTGTGCCCTTCCATATTTA

>DW_B5-2S

ATATTAATCTTCTCCCCCATGATTTATACTGGTGGGTCTCGGGCGAGGCCGTGAATTCAA

GTCTGGTGTCCACTTGTCTCTTTTTTTCTCAGTTGCCTTGATTTAAAAAGGAAACTTTAC

TTCGTACCTGCCTTGTCGGAAATCGACCGCTGCGACGCGCGCGCGGAATAGCATCACCC

>DW_B6-5

GAAAAGACTCATGCGCGGGCGGCCGCGCGTGGCAACGCTCGACCTGGGTATCGCGCGTAC

GACACTGTCGGAAAGGGCGCAGGGCCCACCCACATGTCCAGACCATCCATAAAACTCTGC

CCCGTGATTTCAATAGTGGGGCCTAGTGTCTCCTTCCATATATAAAACTCTGCCCCGTGA

TTTCAACCGGTGGGGGGCCCGCTATGCCTGATTAATTAGCCTCACCCTGATTTCTTTACA

AGGAAACTCACGCTGTCGGAAATCGATCGTTCTAACGCGCGCGCGTAATAGCAT

>DW_A7-7

GAAAAGACTCATACGCGGCCGCACGTGGCAACGCTCGACCTGGGTATCGCGTGTACGACA

CTGTCGGAAAGGGCGCAGGGCCCACCCACATGTCCAGACCATCCATAAAACTCTGCCCCG

TGATTTCAATAGTGGGGCCTAGTGTCTCCTTCCATATATAAAACTCTGCCCCGTGATTTC

AACCGGTGGGGGGCCCGCTATGCCTGATTAATTAGCCTCACCCTGATTTCTTTACAAGGA

AACTTGAGTTTCTAACACGCTGTCGGAAATCGATCGTTCTAACGCGCGCGCGCGTAATAG

CATCCCT

>AT_D2-2

TACGCGAGCGTGCGTTAGAACGATCGATTTCCGACAGCGTGTTAGAAACTCAAGTTTCCT

TGTAAAGAAATCAGGGTGAGGCTAATTAATCAGGCATAGCAGGCCCCCATCGGTTGAAAT

CACGGGGCAGACTTTTATATATGGAAGGAGACACTAGGCCCCACCATTGAAATCACGGGG

CAGAGTTTTATGGATGGTCTGGACATGTGGGTGGGTCTTGCGCCCTTTCCGACAGTGTCG

TACGCGCGATGCCCAGGTCGAGCGTTGCAACGCGCGGCCGCGTATGAGCCTTTTCGC

>AT_D3-2

GGATGCTATTACCCGCGCGCGCGTTAGAACGATCGATTTCCGACAGCGTGTTAGGAACTC

AAGTTTCCTTGTAAAGAAATCAGGGTGATGCTAATTAATCAGGCATAGCGGGCCCCCCAC

CGGTTAAAATCACGGGGCAGAGTTTTATATATGGAAGGAGACAATAGGCCCCACCATTGA

AATCACGGTCAGAATTTTGTGGATGGTCTGGACATGTGGGTGGACCCTGCGCCCTTTCCG

ACAGTGTCGTACGCGCGATGCCCAGGTCGGGCGTTGCAACGCGCGGCCGCGTGTGAGCCT

TTTCGC

>AT_D4-3

AAAAGGCTTCTTCGCGGCAGCGCGTCGCAACGCGCGATTAGCTTAAGGCATTGGATGCTT

TCCTACAGGCACAAGGGGGCCCACCACATGTGTCTTCAACATCAAAAAATCACCCCCCGT

GATTTCAACCGGTGGGGCCGGGGTGCGCCTTCCATATTTTTATCTCCCCCCGTGATTTCA

ACTGGTGGGGCCGGGGTGTTTCCTTTTCTATCTCTCATCCTGGAGTATTTAAAATGGAAA

GTTTACTTCGTAGCTCCTGTCGGAAATCGCTCATCGCAACGAGCGCGCGCGGAATAGCAT

CCCT

>AT_D4-4

GGATGCTATTCCGCGCGTGCTCGTTGCGACGAGCGATTTCCGACAGGAGCTACGAAATAA

ACTTTCCATTTTAAATAAGCCAGGATGAAAGATAGAAAAGGAAACACCTCGGCCCCACTA

GTTGAAATCATGGGGGAAGATAAAAGTATGGAAGGCACACCCCGGCCCCATCAGTTGTAA

TAGGGGGGGGGGTTGTGATTTTTTGATGTTGAAGACACATGTGGTGGGCCCCCTTCTCCC

TGTCCGAAAGCATGCAACGCCTGAGGCTAATTGCGCGTTGCGAGGCGCGGCCGCGAAGGA

GCCTTTTCC

>AT_D2-4S

CTCTAATCCTGGCTTATTTAAAATGGAAAGTTTACTTCCTAAGCTCTTGTCGGACATCGC

TCGTCCCAACGAGCGAGCGCGGAATAGCATCCC

>TU_A5-13

GAAAATGCTCATACGCGGCTGCGCGTTGCAACGCTCGACCACATGGCATCGCGCGTTCGT

GGCATCGCGCGTACGACACTGTCGGAAAGGGCGCAGGGCCCACCCACATGTCCAGACCAT

CCACAAAACTCTGCCCCGTAATTTCAATGGTGGGGCCTAGTGTCTCCTTCCATATATAAA

ACTCTGCCCCGTGATTTCAACCGGTGGGGGGCCCGCTATGCCTGATTAATTAGCATCACC

CTGATTTCTTTACAAGGAAACTTGAGTTCCGAACACGCTGTCGGAAATCGATCGTTCTAA

CGCGCGCGCGCGTAAT

>TU_A5-0S

AAAAGGCTCCATTGCGGCCGCGCGTCCCGCGATTAGCCTCATGCGGTACATGCTTTCGAA

CATGGACAAAAGGGCCCAACACATGTGTCTTCAACATCAAAAAATCACCCCCACGATTTC

AACTGGTGGGGCCGAGTGTGCCCTTCCATATTTAAAATGGAAAGTTTACATCGTAGGCTC

TTGTCGGAAATCGTTGGTCGCAACAAGCGTGTGCAGGATAACATCCCT

>TU_A6-3

GGATGCTATTACGCGCGCGCGCGTTAGAACGATCGATTTCCGACAGCGTGTTAGAAACTC

AAGTTTCCTTGTAAAGAAATCAGGGTGAGACTAATTAATCAGGCATAATGGGCCCCCCAC

CGGTTGAAATCACGGGGCAGAGTTTTATATATGGAAGGAGACACTAGGCCCCACCATTGA

AATCACGGGGCAGAGTTTTATGGATGGTCTGGACATGTGGGTGGGCCCTGCGTTCTTTCC

GACAGTGTCGTACGCGCGATGCCCAGGTCGAGCGTTGCAACGCGCGGCCGCGTATGAGTC

TTTTCGC

>WE_B1-4

AGGGATGCTATTACGCGCGCGCGCGTTAGAACGATCGATTTCCGACAGCGTGTTAGAAAC

TCAAGTTTCCTTGTAAAGAAATCAGGGTAAGGCTAATTAATCAAGCATAGCGGGCTCCCC

AGTGGTTGAAATCACGGGGCAGAGTTTTATATATGGAAGGAGACATTAGGCCCCACTATT

GAAATCACGGGGCTTTTATGGATGGTCTGGACATGTGGGTGGGCCCTGCGCCCTTTCCGA

CAGTGTCGTACGCGCTGTGCCCAGGTCGAGCGTTGCCACGCGCGGCCGCGTATGAGTCTT

TTCGC

>WE_B2-5

GAAAAGACTTATACGCGGCCGCGCGTTGCAACGCTCAACCTGGGCATCGCGCGTACGACA

CTGTCGGAAAGGGCACAGGACCCACCCACATGTCCAGACCATCCATAAAACTATGCCCCG

TGATTTCAATGGTGGGGCCTAGTGTCTCCTTCCATATATAAAACTCTGCCCCGTGATTTC

AACCGGTGGGGGGCCCGCTATGCCTGATTAATTAGCCTCACCCTGATTTCTTACAAGGAA

ACTTGAGTTTCTAACGCGCTGTCGGAAATCGATCGTTCTCGCACGCGCGCGTAATAGT

>WE_B2-3

AAGGCGGGATGAGAGATAGAAAAGGAAACATCCCAGCCCCACCAGTTGAAATCACGGAGG

GACATAAAAATATGAAAGGCACACCCCGACCCCACCAGTTGAAATCATGGGGGTTTTTTG

ATATTGAAGACACATGCGGTGGGCCCCTTTGTCCCTGTCCAAAAGCATGCAACGCCTGAA

GCTAATCCCGCGTTGCGACGCGCGGCCGCGAAGAAGCCTTTTCG

>WE_A4-1

AGGCTCCTTCGCGGCCGCGCGTCGCAACGCGCGATTAGCCTCGGGCGTTGTGTGCTTTCG

GACAGGGACAAACGGGCCCACCACATGTGTCTTCAACATCAAGAAAATCAACCCCCGTGA

TTTCAACTGGTGGGGCCGGGGTGTGCCTTCCATATTTTTTATCTCCCTCCGTGATTTGAA

CTGATGGGGCTGGGATGTTTCCTTTTCTATCTCTAATCCTGGCTTATTTAAAATGGAAAG

TTTACTTCATACCTCTTGTCGGAAATCGCTCGTCGCAACGAGCGCGCGCGGAATAGCATC

G

>WE_A4-5

AGGCTCCTTCGCGGCCGCGCGTCGCAACGCGCGATTAGCCTCAGGCGTTGTGTGCTTTCG

GACAGGGACAAACGGGCCCACCACATGTGTCTTCAACATCAAGAAAATCAACCCCCGTGA

TTTCAACTGGTGGGGCCGGGGTGTGCCTTCCATATATTTTATCTCCCTCCGTGTTTTGAA

CTGATGGGGCTGGGATGTTTCCTTTTCTATCTCTAATCCTGGCTTATTTAAAATGGAAAG

TTTACTTCATACCTCTTGTCGGAAATCGCTCGTCGCAACGAGCGCGCGCGGAATAGCATC

A

>WE_B3-4

GTGGGCCAGTAGGTCGAAGCCTAAGAAGGCGTTGGATTTACATCCAATGGTCAGCATTCT

TCTTAAATCGCTCGTCTTCTTCCCCCAGCGCTGCCGGAACCGCCTGCTCCAGCCTTTGGC

GTCCAGCGGTGTTGTGTTGCACTCCTCAGCAAAAAGCTACCCCGCCGACGGCCAGGCCAT

CCCTCCACTCTGCACCTCCTGTTATTCTCTGCCGAGTCTAGGCCCACCCCGCACCCAAAC

CAGTCAAGTTTCCCACTCCTCTCCGTCTGCGACTCCATTGCCGCGTCTTCCCCATCT

>WE_B4-6

GAAAAGACTCATACGCGGCCGCGCGTGGCAACGCTCGACCTGGGCACAGCGCGTACGACA

CTGTTAGAAAGGGCGCAGGGCCCACCCACATGTCCAGACCATCCATAAAACTCTGCTCCG

TGATTTCAATAGTGGGGCCTAATGTCTCCTTCCATATATAAAACTCTGCCCCGTGATTTC

AACCAGTGGGGGCCCCGCTATGCTTGATTAATTAGCCTTACCCTGATTTCTTTACAAGGA

AACTTGAGTTTCTAACACGCTGTCGGAAATCGATCGTTCTAACGCGCGCGCGT

>WE_A5-6

GAAAAGACTCATACGCGGCCGCGCGTGGCAACGCTCGATCTGGGTATCGCGCGTACGACA

CTGTCGGAAAGGGCGCAGGGCCCACCCACATGTCCAGACCATCCATAAAACTCTACCCCG

TGATTTCAATAGTGGGGCCTAGTGTCTCCTTCCATATATAAAACTCTGCCCCGTGATTTC

AACCGGTGGGGGGCTCGCTATGCCTGATTAATTAGCCTCACCCTGAGTTCTTTACAAGGA

AACTTGAGTTTCTAACACGCTGTCGGAAATCGATCGTTCTAACGCGCGCGTAATAGCATC

CCTCT

>WE_A5-3S

AAAAGGCTCCATTGCGGCCGCGCGTCCCGCGATTAGCCTCATGCGGTACATGCTTTCGAA

CATGGACAAAAGGGCCCAACACATGTGTCTTCAACATCAAAAAATCACCCCCACGATTTC

AACTGGTGGGGCCGAGTGTGCCCTTCCATATTTA

>WE_B5-3S

CAACTTTTGGGGCCTGGGCGTGTAATTCCATATTAATCTTCTCCCCCATGATTTATACTG

GTGGGTCTCGGGCGAGGCCGTGAATTCAAGTCTGGTGTCCACTTGTCTCTTTTTTTCTCA

GTTGCCTTGATTTAAAAAGGAAACTTTACTTCGTACCTGCCTTGTCGGAAATCGACCGCT

GCGACGCGCGCGCGGAATAGCATCACCC

>WE_B5-5S

GTGATTTTTTTGATGTTGAAGACACATGTGGTGGGCCCGTTTGTCCCTGTCCGAAAGCAC

ACCACGCCTGAGGCTAATCGCGCGTTGCGACGCGCGGCCGCGAAGGAGCCTTTTCGT

>WE_B5-6

GAAAAGACTCATACGCGGCCGCGCGTGGCAACGCTCGACCTGGGCACAGCGCGTACGACA

CTGTCGGAAAGGGCGCAGGGCCCACCCACATGTCCAGACCATCCATAAAAGCCCCGTGAT

TTCAATAGTGGGGCCTAATGTCTCCTTCCATATATAAAACTCTGCCCCGTGATTTCAACC

ACTGGGGGGCCCGCTATGCTTGATTAATTAGCCTTACCCTGATTTCTTTACAAGGAAACT

TGAGTTTCTAACACGCTCGATCGTTCTAACGCGCGCGCGTAATAGCATCCCTCG

>WE_B5-4

GCTATTACGCGCGCGCGTTAGAACGATCGATTTCCGACAGCGTGTTAGAAACTCAAGTTT

CCTTGTAAAGAAATCAGGGTGAGGCTAATTAATCAGGCATAGCGGGCCCCCCACCGGTTG

AAATCACGGGGCAGAGTTTTATATATGGAAGGAGACACTAGGCCCCACTATTGAAATCAC

GGGGCAGAGTTTTATGGATGGTCTGGACATGTGGGTGGGCCCTGCGCCCTTTCCGACAGT

GTCGTACGCGTGATACCCAGGTCGAGCGTTGCCACGCGCGGCCGCGTATGAGTCTTTTCG

T

>WE_A6-2

GAAAAGACTCATACGCGGCCGCGCGTGGCAACGCTCGACCTGGGTATCGCGCGTACGACA

CTGTCGGAAAGGGCGCAGGGCCCACCCACATGTCCAGACCATCCATAAAACTCTGCCCCG

TGATTTCAATAATGGGGCCTAGTGTCTCCTTCCATATATAAAACTCTGCCCCGTGATTTC

AACCGGTGGGGGGCACGCTATGCCTGATTAATTAGCCTCATCCTGATTTCTTTACAAGGA

AACTTGAGTTTCTAACACGCTGTCAGAAATCGATCGTCTAACGCGCGCGCGCGTAATAGC

ATCCCTC

>WE_A7-3

GGATGCTATTACGCGCGCGCGCGTTAGAACGATCGATTTCCGACAGCGTGTTAGAAACTC

AAGTTTCCTTGTAAAGAAATCAGGGCGAGGCTAATTAATCAGGCATAGCGGGCCCCCCAC

CGGTTGAAATCACGGGGCAGAGTTTTATATATGGAAGGAGACACTAGGCCCCACTATTGA

AATCACGGGGCAGAGTTTTATGGATGGTCTGGACATGTGGGTGGGCCCTGCGCCCTTTCC

GACAGTGTCGTACGCGCGATACCCAGGTCGAGCGTTGCCACGCGCGGCCGCGTATGAGTC

TTTTCGA

>WE_A7-4

GAAAAGACTCATACGCGGCCGCGCGTGGCAACGCTCCACCTGGGTATCGCGCGTACGACA

CTGTCGGAAGGGCGCAGGGCCCACCCACATGTCCAGACCATCCATAAAACTCTGCCCCGT

GATTTCAATAGTGGGGCCTAGTGTCTCCTTCCATATATAAAACTCTGCCCCGTGATTTCA

ACCGGTGGGGGCCCGCTATGCCTGATTAATTAGCCTCACCCTGATTTCTTTACAAGGAAA

CTTGAGTTTCTAACACGCTGTCGGAAATCGATCGTTCTAACGCGCGCGCGCGTAATAGCA

TCCCT

>WE_A7-8

GAAAAGACTCATACGCGGCCGCACGTGGCAACGCTCGACCTGGGTATCGCGTGTACGACA

CTGTCGGAAAGGGCGCAGGGCCCACCCACATGTCCAGACCATCCATAAAACTCTGCCCCG

TGATTTCAATAGTGGGGCCTAGTGTCTCCTTCCATATATAAAACTCTGCCCCGTGATTTC

AACCGGTGGGGGGCCCGCTATGCCTGATTAATTAGCCTCACCCTGATTTCTTTACAAGGA

AACTTGAGTTTCTAACACGCTGTCGGAAATCGATCGTTCTAACGCGCGCGCGCGTAATAG

CATCCCT

>WE_A7-5

GAAAAGGCTCCTTCGCGGCCGCGCGTCGCAACGCGCGATTAGCCTCAGGCGTTGTGTGCT

TTCGGACAGGGACAAACGGGCCCACCACATGTGTCTTCAACATCAAGAAAATCAACCCCC

GTGATTTCAACTGGTGGGGCCGGGGTGTGCCTTCCATATTTTTTATCTCCCTCCGTGATT

TGAACTGATGGGGCTGGGATGTTTCCTTTTCTATCTCTAATCCTGGCTTATTTAAAATGG

AAAGTTTACTTCGTACCTCTTGTCGGAAATCGCTCGTCGCAACGAGCGCACGCGGAATAG

CATCCCT

>WE_B7-4

TGCTATTCCGCGCGCTCGTTGCGACGAGCGATTTCCGACAAGAGGTATGAAGTAAACTTT

CCATTTTAAATAAGCCAGGATTAGAGATAGAAAAGGAAACATCCCAGCCCCATCAGTTCA

AATCACGGAGGGAGATAAAAAATATGGAAGGCACACCCCGGCCCCACCAGTTGAAATCAC

GGGGGTTGATTTTCTTGATGTTGAAGACACATGTGGTGGGCCCGTTTGTCCCTGTCCGAA

AGCACACAACGCCTGAGGCTAATCGCGCGTTGCGACGCGCGGCCGCGAAGGAGCCTTTTC

GG

>WE_B7-6

GAAAAGGCTCCTTCGCGGCCGCGCGTCGCAACGCGCGATTAGCCTCAGGCGTTGTGTGCT

TTCGGACAGGGACAAACGGGCCCACCACATGTGTCTTCAACATCAAGAAAATCAACCCCC

GTGATTTCAACTGGTGGGGCCGGGGTGTGCCTTCCATATTTTTTTTATCTCCCTCCGTGT

TTTGAAGTGATGGGGCTGGGATGTTTCCTTTTCTATCTCTAATCCTGGCTTATTTAAAAT

GGAAAGTTTACTTCATACCTCTTGTCGGAAATCGCTCGTCGCAACGAGCGCGCGCGGAAT

AGCG

>WE_A2-MH

cgaaaaggctccttcgcggccgcgcgtcgcaacgcgcgattagcctcaggcgttgtgtgc

tttcggacagggacaaacgggcccaccacatgtgtcttcaacatcaagaacaacccccgt

gatttcaactggtggggccggggtgtgccttccatattttttatctccctccgtgatttg

aattgatggggctgggatgtttccttttctatctctaatcctggcttatttaaaatggaa

agtttacttcatacctcttgtcggaaatcgctcgtcgcaacgagcgcgcgcggaatagca

tcccctc
