# Supplementary material for: The Evolutionary Dynamics of a Novel Miniature Transposable Element in the Wheat Genome
Source: Front Plant Sci. 2020 Jul 31;11:1173. doi: 10.3389/fpls.2020.01173 (PMC7438880; doi:10.3389/fpls.2020.01173)
Supplement: Supplementary file 1 [file DataSheet_1.zip › New folder (2)/Data 2.DOCX]

***Mariam_2_insertions***

>Mariam2_concensus

cgaaagagctccttcacgggcgcgcgtcgcaacgcgcgaccttcctttcatgtatgtgatgccgacaaggtctggtacaggcccaccatgtggccaagaagtcaaacgtgcagaggcatgcacgggagccgctggcacatgcatctctctcgtagtataattttctcccccctgattttcacgggtggggcccggtgtgtttcctaatcatgctaacctgatttgccaaaaaaggaaggttcgctcatgtcggaaatcgatcgtgccaacgcgcgcgcgcgagatagcatc

>TA_A2-1

cgaaagagctccttcacgggcgcgcgtcgcaacgcgcgaccttcctttcatgtatgtgatgccgacaaggtctggtacaggcccaccatgtggccaagaagtcaaacgtgcagaggcatgcacgggagccgctggcacatgcatctctctcgtagtataattttctcccccctgattttcacgggtggggcccggtgtgtttcctaatcatgctaacctgatttgccaaaaaaggaaggttcgctcatgtcggaaatcgatcgtgccaacgcgcgcgcgcgagatagcatc

>WE_A2-2

cgaaagagctccttcacgggcgcgcgtcgcaacgcgcgaccttcctttcatgtatgtgatgccgacaaggtctggtacaggcccaccatgtggccaagaagtcaaacgtgcagaggcatgcacgggagccgctggcacatgcatctctctcgtagtataattttctcccccctgattttcacgggtggggcccggtgtgtttcctaatcatgctaacctgatttgccaaaaaaggaaggttcgctcatgtcggaaatcgatcgtgccaacgcgcgcgcgcgagatagcatc

>WE_A3-2

cgaaagagctccttcacgggcgcgcgtcgcaacgcgcgaccttcctttcatgtatgtgatgccgacaaggtctggtacaggcccaccatgtggccaagaagtcaaacgtgcagaggcatgcacgggagccgctggcacatgcatctctctcgtagtataattttctcccccctgattttcacgggtggggcccggtgtgtttcctaatcatgctaacctgatttgccaaaaaaggaaggttcgctcatgtcggaaatcgatcgtgccaacgcgcgcgcgcgagatagcatc

>TA_A4-7

cgaaagagctccttcacgggcgcgcgtcgcaacgcgcgaccttcctttcatgtatgtgatgccgacaaggtctggtacaggcccaccatgtggccaagaagtcaaacgtgcagaggcatgcacgggagccgctggcacatgcatctctctcgtagtataattttctcccccctgattttcacgggtggggcccggtgtgtttcctaatcatgctaacctgatttgccaaaaaaggaaggttcgctcatgtcggaaatcgatcgtgccaacgcgcgcgcgcgagatagcatc

>DW_A4-8

cgaaagagctccttcacgggcgcgcgtcgcaacgcgcgaccttcctttcatgtatgtgatgccgacaaggtctggtacaggcccaccatgtggccaagaagtcaaacgtgcagaggcatgcacgggagccgctggcacatgcatctctctcgtagtataattttctcccccctgattttcacgggtggggcccggtgtgtttcctaatcatgctaacctgatttgccaaaaaaggaaggttcgctcatgtcggaaatcgatcgtgccaacgcgcgcgcgcgagatagcatc

>WE_A4-9

cgaaagagctccttcacgggcgcgcgtcgcaacgcgcgaccttcctttcatgtatgtgatgccgacaaggtctggtacaggcccaccatgtggccaagaagtcaaacgtgcagaggcatgcacgggagccgctggcacatgcatctctctcgtagtataattttctcccccctgattttcacgggtggggcccggtgtgtttcctaatcatgctaacctgatttgccaaaaaaggaaggttcgctcatgtcggaaatcgatcgtgccaacgcgcgcgcgcgagatagcatc

>TA_A4-10

cgaaagagctccttcacgggcgcgcgtcgcaacgcgcgaccttcctttcatgtatgtgatgccgacaaggtctggtacaggcccaccatgtggccaagaagtcaaacgtgcagaggcatgcacgggagccgctggcacatgcatctctctcgtagtataattttctcccccctgattttcacgggtggggcccggtgtgtttcctaatcatgctaacctgatttgccaaaaaaggaaggttcgctcgtgtcggaaatcgatcgtgccaacgcgcgcgcgcgagatagcatc

>DW_A4-11

cgaaagagctccttcacgggcgcgcgtcgcaacgcgcgaccttcctttcatgtatgtgatgccgacaaggtctggtacaggcccaccatgtggccaagaagtcaaacgtgcagaggcatgcacgggagccgctggcacatgcatctctctcgtagtataattttctcccccctgattttcacgggtggggcccggtgtgtttcctaatcatgctaacctgatttgccaaaaaaggaaggttcgctcgtgtcggaaatcgatcgtgccaacgcgcgcgcgcgagatagcatc

>DW_A4-3S

GATGCTATCTCGCATGCGTGCGCTGGCACGTTCGATTTCCGACAAGAGCAAACCTTCCTTTTTTGCAAATCAGGTTAGCAATATTAGGAAACACACCGGGCCCCACCCCGTTAAATTCAGAGGGGGGAGAGAAAATTATACTTACGAGAGAGATGCATGTGACAGCGGCCCCGGTGCATG

>WE_A5-2

cgaaagagctccttcacgggtcgcgcgtcaccttcctttcatgtatgtgatgccgacaaggtctggtacaggcccaccatgtggccaaaaagtcaaacgtgcagaggcatgcacgggagccgctggcacatgcatctctctcgtactataattttctcccccctgatttTcacggggtggggggtggggcctggtgtgtttcctaatcatgctaacctgatttgccaaaaaaggaaggttcgctcgtgtcggaaatcgatcgtgccaacgcgcgcgcgcgagatac

>DW_A5-7

cgaaagagctccttcacgggcgcgcgtcgcaacgcgcgaccttcctttcatgtatgtgatgccgacaaggtctggtacaggcccaccatgtggccaagaagtcaaacgtgcagaggcatgcacgggagccgctggcacatgcatctctctcgtagtataattttctcccccctgattttcacgggtggggcccggtgtgtttcctaatcatgctaacctgatttgccaaaaaaggaaggttcgctcatgtcggaaatcgatcgtgccaacgcgcgcgcgcgagatagcatc

>TA_A5-8S

TGCTATCTCGCGCACGCTCGTTGAGACACTCGGTTTCCGACAAAGTAAACTTTCCTGTTTAAGCAAATAAGCGTGAGGTGGAAATGGAAAGACAGGTTGGGCCCCACCCGTTAAATTCAGCAGGGGAGAGAGATTAT

>WE_A5-9S

TGCTATCTCGCGCACGCTCGTTGAGACACTCGGTTTCCGACAAAGTAAACTTTCCTGTTTAAGCAAATAAGCGTGAGGTGGAAATGGAAAGACAGGTTGGGCCCCACCCGTTAAATTCAGCAGGGGAGAGAGATTAT

>DW_A5-10S

TGCTATCTCGCGCACGCTCGTTGAGACACTCGGTTTCCGACAAAGTAAACTTTCCTGTTTAAGCAAATAAGCGTGAGGTGGAAATGGAAAGACAGGTTGGGCCCCACCCGTTAAATTCAGCAGGGGAGAGAGATTAT

>TU_A5-11S

TGCTATCTCGCGCACGCTCGTTGAGACACTCGGTTTCCGACAAAGTAAACTTTCCTGTTTAAGCAAATAAGCGTGAGGTGGAAATGGAAAGACAGGTTGGGCCCCACCCGTTAAATTCAGCAGGGGAGAGAGATTAT

>TU_A6-4S

TAATCTTCGCCCCCCCTGATTTCAATGGGTGGGGCCCGGGGTGGGGCCTCGATTTACCTTAAATGACATTCCAGACTTGCTAAAAAAAGCAAAGTGCAGCCTGTCGGAAATCGCGCGTTCCAGCGCGCGCGCGCGAGATAGCA

>TA_A7-1

cgaaagagctccttcacgggcgcgcgtcgcaacgcgcgaccttcctttcatgtatgtgatgccgacaaggtctggtacaggcccaccatgtggccaagaagtcaaacgtgcagaggcatgcacgggagccgctggcacatgcatctctctcgtagtataattttctcccccctgattttcacgggtggggcccggtgtgtttcctaatcatgctaacctgatttgccaaaaaaggaaggttcgctcatgtcggaaatcgatcgtgccaacgcgcgcgcgcgagatagcatc

>WE_A7-2

cgaaagagctccttcacgggcgcgcgtcgcaacgcgcgaccttcctttcatgtatgtgatgccgacaaggtctggtacaggcccaccatgtggccaagaagtcaaacgtgcagaggcatgcacgggagccgctggcacatgcatctctctcgtagtataattttctcccccctgattttcacgggtggggcccggtgtgtttcctaatcatgctaacctgatttgccaaaaaaggaaggttcgctcatgtcggaaatcgatcgtgccaacgcgcgcgcgcgagatagcatc

>TA_D6-1

cgaaagagctccttcacgggcgcgcgtcgcaacgcgcgaccttcctttcatgtatgtgatgccgacaaggtctggtacaggcccaccatgtggccaagaagtcaaacgtgcagaggcatgcacgggagccgctggcacatgcatctctctcgtagtataattttctcccccctgattttcacgggtggggcccggtgtgtttcctaatcatgctaacctgatttgccaaaaaaggaaggttcgctcatgtcggaaatcgatcgtgccaacgcgcgcgcgcgagatagcatc

>DW_B2-7

cgaaagagctccttcacgggcgcgcgtcgcaacgcgcgaccttcctttcatgtatgtgatgccgacaaggtctggtacaggcccaccatgtggccaagaagtcaaacgtgcagaggcatgcacgggagccgctggcacatgcatctctctcgtagtataattttctcccccctgattttcacgggtggggcccggtgtgtttcctaatcatgctaacctgatttgccaaaaaaggaaggttcgctcatgtcggaaatcgatcgtgccaacgcgcgcgcgcgagatagcatc

>TA_B4-1S

CGAAAGAGCTCCTTCACGGGCGCGCGTCGCAACGCGCGACCTTCCTTTCATGTATGTGATGCCGACAAGGTCTGGTACAGGCCCACTATGTGGCCAAGAAGTCAAACGTGCAGAGGCATGCACGGGAGCCGCTGGCACATGCATCTCTCTCGTAGTATAATTTTCTCCCCCCCTGATTTTCACGGGTGGGGCCCGGTGTGGTT

>DW_B4-2S

CGAAAGAGCTCCTTCACGGGCGCGCGTCGCAACGCGCGACCTTCCTTTCATGTATGTGATGCCGACAAGGTCTGGTACAGGCCCACCATGTGGCCAAGAAGTCAAACGTGCAGAGGCATGCACGGGAGCCGCTGGCACATGCATCTCTCTCGTAGTATAATTTTCTCCCCCCTGATTTTCACGGGTGGGGCCCGGTGTGGTT

>WE_B4-3S

CGAAAGAGCTCCTTCACGGGCGCGCGTCGCAACGCGCGACCTTCCTTTCATGTATGTGATGCCGACAAGGTCTGGTACAGGCCTACCATGTGGCCAAGAAGTCAAACGTGCAGAGGCATGCACGGGAGCCGCTGGCACATGCATCTCTCTCGTAGTATAATTTTCTCCCCCCTGATTTTCACGGGTGGGGCCCGGTGTGGTT

>TA_B5-8S

TCTCTCCCCCGCTGAATTTAACGGGTGGGGCCCAACCCGTCTTTCCATTTCCACCTCACGCCCATTTGCTTAAACAGGAAAGTTTACTTTGTCAGAAACCGAGCGTCTTAACGAGCGCGCGCGAGATAGCA

>DW_B5-9S

TCTCTCCCCCGCTGAATTTAACGGGTGGGGCCCAACCCGTCTTTCCATTTCCACCTCACGCCCATTTGCTTAAACAGGAAAGTTTACTTTGTCGGAAACCGAGCGTCTTAACGAGCGCGCGCGAGATAGCA

>WE_B5-10S

TCTCTCCCCCGCTGAATTTAACGGGTGGGGCCCAACCCGTCTTTCCATTTCCACCTCACGCCCATTTGCTTAAACAGGAAAGTTTACTTTGTCGGAAACCGAGCGTCTTAACGAGCGCGCGCGAGATAGCA

>DW_B6-1

cgaaagagctccttcacgggcgcgcgtcgcaacgcgcgaccttcctttcatgtatgtgatgccgacaaggtctggtacaggcccaccatgtggccaagaagtcaaacgtgcagaggcatgcacgggagccgctggcacatgcatctctctcgtagtataattttctcccccctgattttcacgggtggggcccggtgtgtttcctaatcatgctaacctgatttgccaaaaaaggaaggttcgctcatgtcggaaatcgatcgtgccaacgcgcgcgcgcgagatagcatc

>WE_B6-2

cgaaagagctccttcacgggcgcgcgtcgcaacgcgcgaccttcctttcatgtatgtgatgccgacaaggtctggtacaggcccaccatgtggccaagaagtcaaacgtgcagaggcatgcacgggagccgctggcacatgcatctctctcgtagtataattttctcccccctgattttcacgggtggggcccggtgtgtttcctaatcatgctaacctgatttgccaaaaaaggaaggttcgctcatgtcggaaatcgatcgtgccaacgcgcgcgcgcgagatagcatc

>WE_B6-3

cgaaagagctccttcacgggcgcgcgtcgcaacgcgcgaccttcctttcatgtatgtgatgccgacaaggtctggtacaggcccaccatgtggccaagaagtcaaacgtgcagaggcatgcacgggagccgctggcacatgcatctctctcgtagtataattttctcccccctgattttcacgggtggggcccggtgtgtttcctaatcatgctaacctgatttgccaaaaaaggaaggttcgctcatgtcggaaatcgatcgtgccaacgcgcgcgcgcgagatagcatc

>DW_U

cgaaagagctccttcacgggcgcgcgtcgcaacgcgcgaccttcctttcatgtatgtgatgccgacaaggtctggtacaggcccaccatgtggccaagaagtcaaacgtgcagaggcatgcacgggagccgctggcacatgcatctctctcgtagtataattttctcccccctgattttcacgggtggggcccggtgtgtttcctaatcatgctaacctgatttgccaaaaaaggaaggttcgctcatgtcggaaatcgatcgtgccaacgcgcgcgcgcgagatagcatc

>TA_D5-8S

TCTCCCCCGCTGAATTTAACGGGTGGGGCCCAACCTGTCTTTCCATTTCCACCTCACGCTCATTTGCTTAAACAGGAAAGTTTACTTTGTCGGAAACCGAGCGTCTCAACGAGCGCGCGTGAGATAGCA

>AT_D5-9S

TCTCCCCCGCTGAATTTAACGGGTGGGGCCCAACCTGTCTTTCCATTTCCACCTCACGCTCATTTGCTTAAACAGGAAAGTTTACTTTGTCGGAAACCGAGCGTCTCAACGAGCGCGCGTGAGATAGCA

>AT_D6-2

GAGGCTATCTCGCGCGCGCGCGCGTTGGCGCGGCCGATTTCCGACAGGACTGCTGATTCCCTTTTTGGCAAATCTGATGAGCGAGATTAGTGGTACACACCTGGCCCCACCAGTTAAAATCAGGGGGAGAGAAAATTAGGGAGAGATGCATGCGCTAGAGCTTCTGGCGCACGCCTCGGTAGCGTTGGTCGTTGGCCACATGGTGGGCCTTGCTGCTGACCCTGTCCGCAGAACAGAAAGGAGATGGATTAGATCGCGCGTTGCGACGTGCGCCCGTGAAAGAGTAATTTCG

>AT_D7-1

GAGGCTATCTCGCGCGCGCGCGTTGGCGCGGCCGATTTCCGACAGGACTGCTGATTCCCTTTTTGGCAAATCTGATGAGCGAGATTAGTGGTACACACCTGGCCCCACCAGTTAAAATCAGGGGGGAGAGAAAATTAGGGAGAGATGCATGCGCTAGAGCTTCTGGTGCACGCCTCGGTAGCGTTGGTCGTTGGCCACATGGTGGGCCTTGCTGCTGACCCTGTCCGCAGAATATGATCGCGCGTTGCGACGTGCGCCCGTGAAAGAGTAATTTCG
