# Supplementary material for: The Evolutionary Dynamics of a Novel Miniature Transposable Element in the Wheat Genome
Source: Front Plant Sci. 2020 Jul 31;11:1173. doi: 10.3389/fpls.2020.01173 (PMC7438880; doi:10.3389/fpls.2020.01173)
Supplement: Supplementary file 1 [file DataSheet_1.zip › New folder (2)/Sup Figures.DOCX]

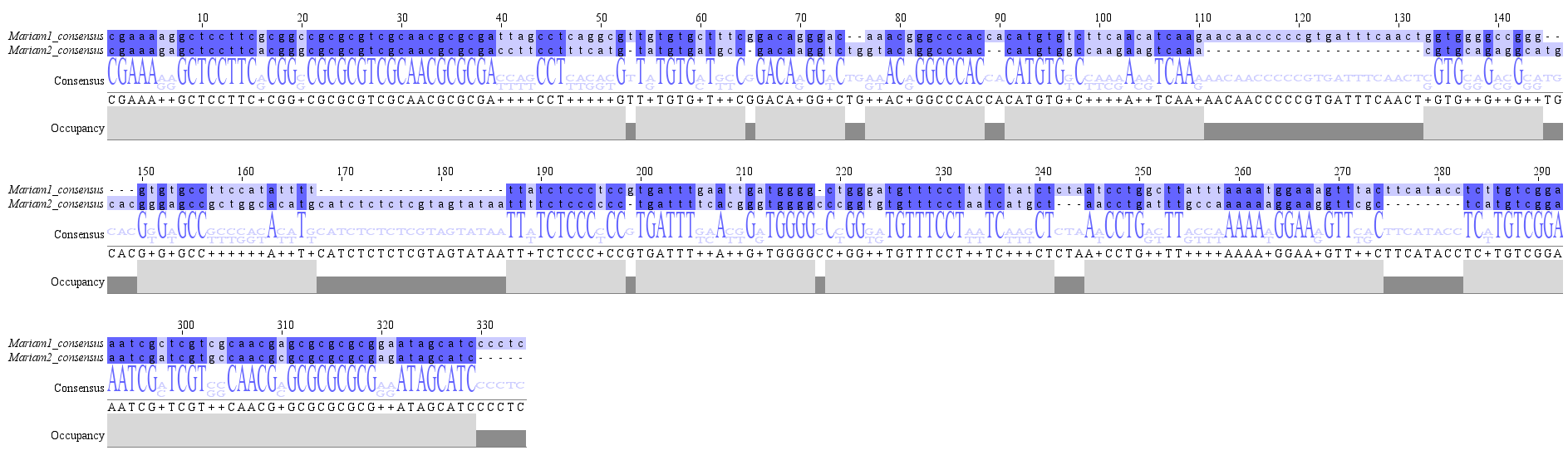


**Supplementary Fig. 1**. Multiple Sequence Alignment of *Mariam1* and *Mariam2* consensus sequences using MAFFT v7 shown in Jalview software. Dark purple represents >90% identity, light purple represents <50% identity.


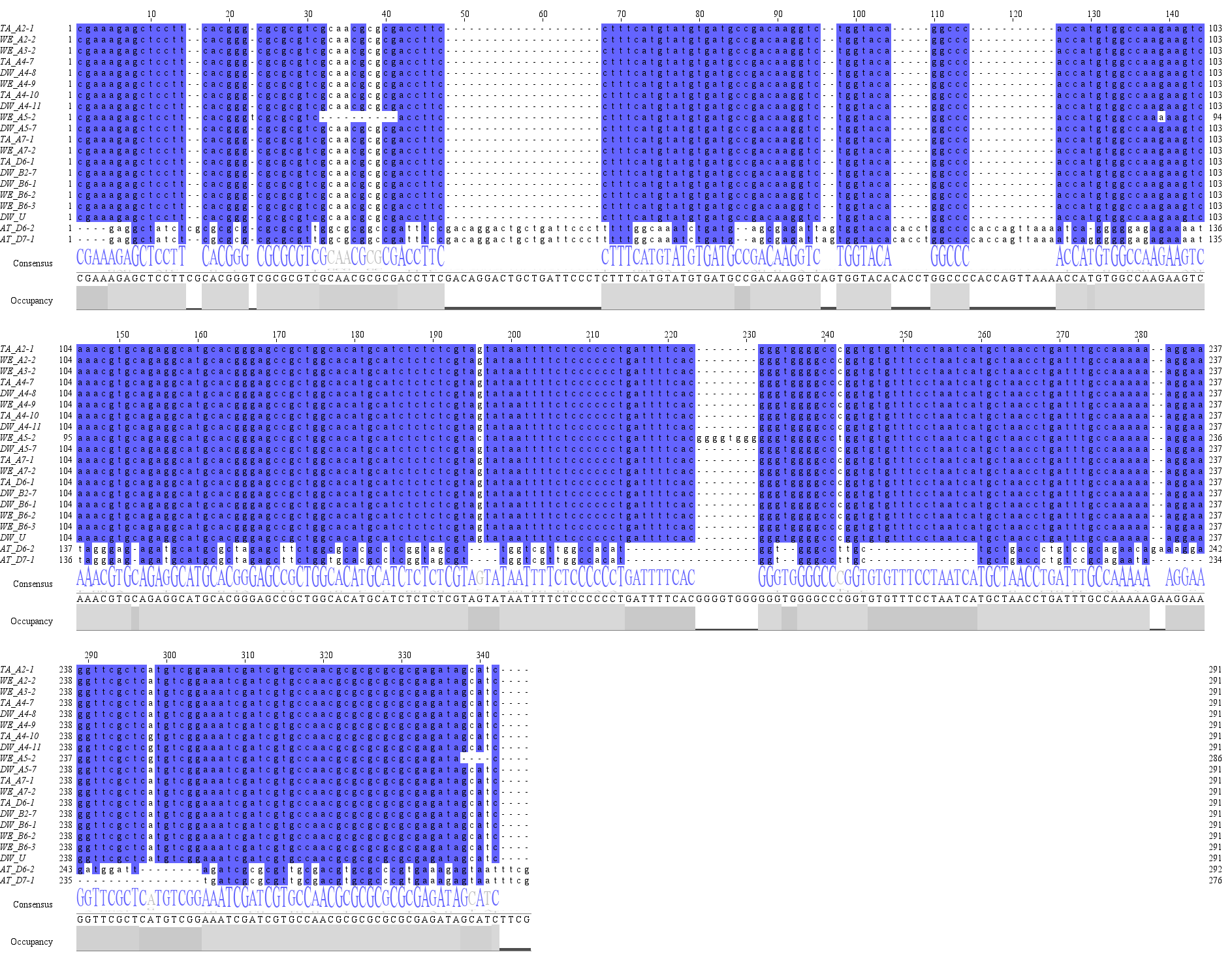


**Supplementary Fig. 2**. Multiple Sequence Alignment of *Mariam2* full-length sequences using MAFFT v7 shown in Jalview software. Dark purple represents >90% identity.


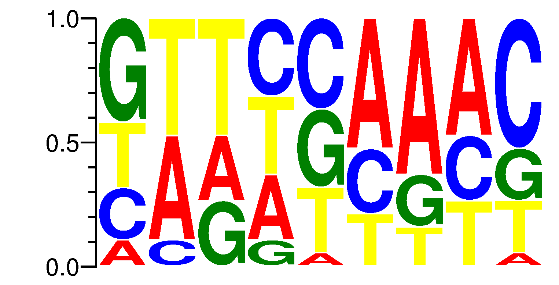


**Supplementary Fig .3. Sequence logo representing target site preference of *Mariam2* sub-family.**Created using WebLogo 3.0 package. The letter height correlates to the probability of each nucleotide at the given position. The sequence logo demonstrated a certain sequence preference at position 7, however the 9-bp duplicated sequences were not conserved.

**a). b).**

**
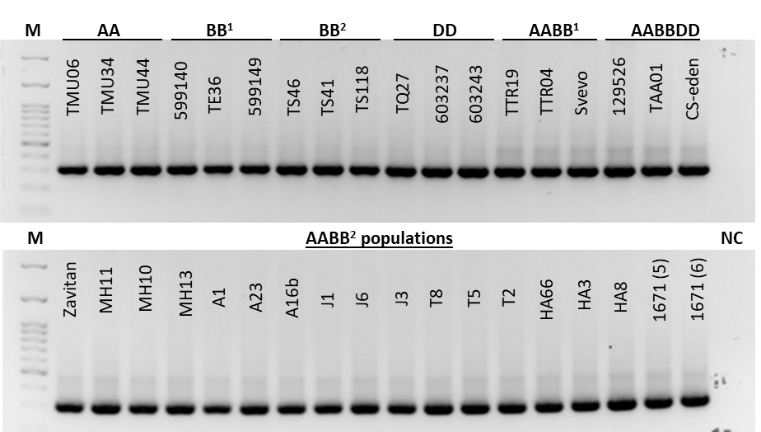

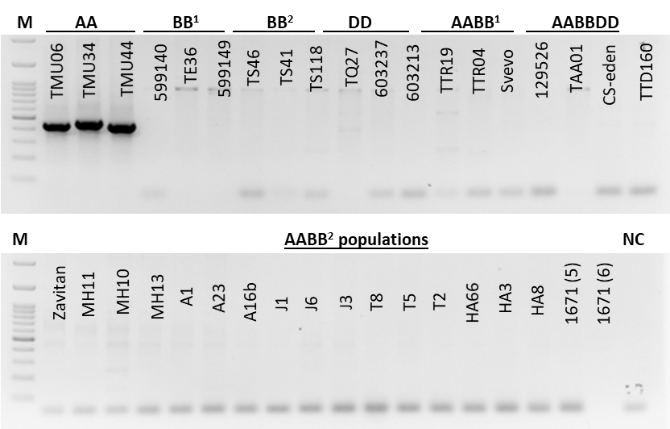
**

**c). d).**

**
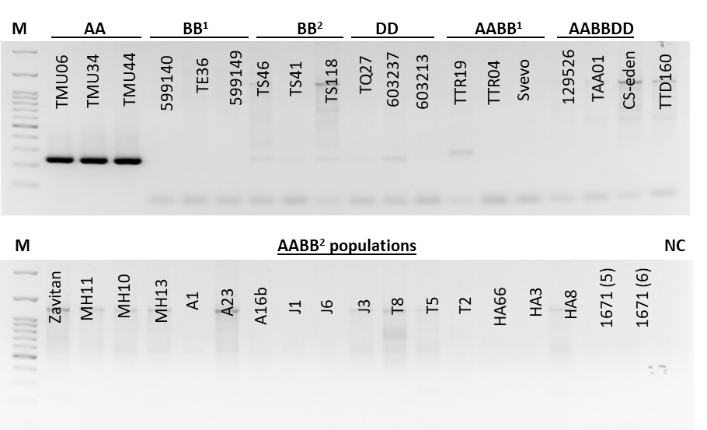

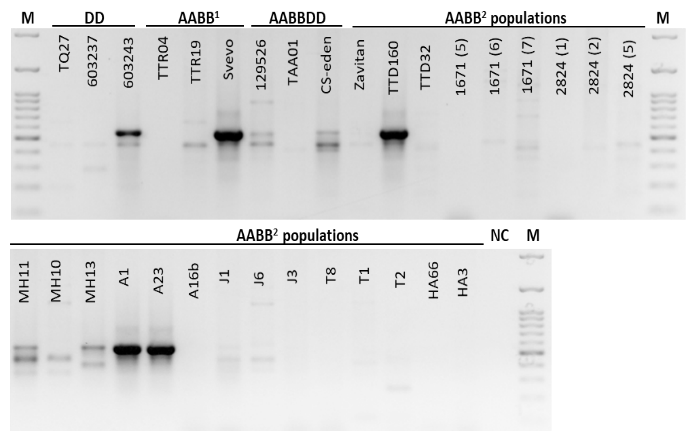
**

**e). f).**

**
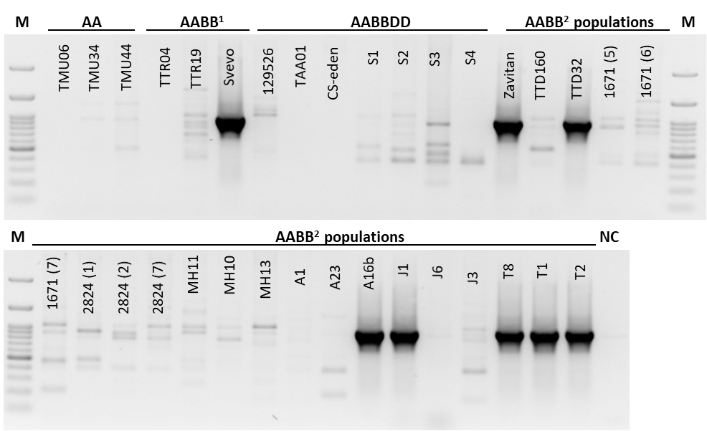

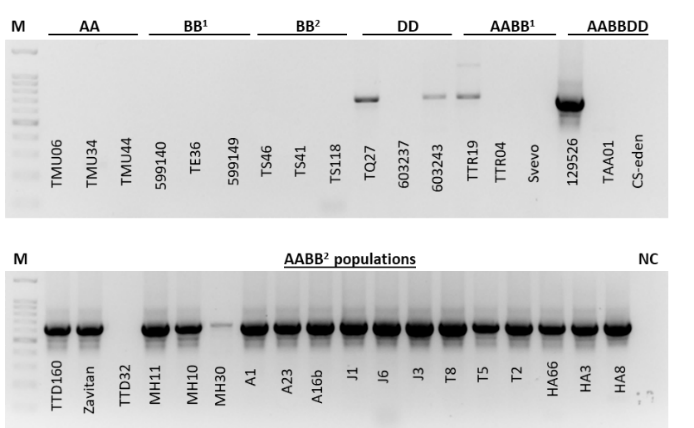
**

**Supplementary Fig .4. Site specific PCR analysis of different *Mariam* insertions.
a.** Insertions **A5-8/9/10S**, **B5-8/9/10S**, **D5-8/9S** within intron 1 of gene TRIDC5AG009460 coding for MUSE14 and all its orthologues. All accessions presented a full site (band size = 315 bp). **b.** Insertion **A6-3** that was found only in TU (*T.urartu*) accessions (band size = 470 bp) . **c.** Insertion **A6-4S** that was found only in TU accessions (band size = 245 bp). **d.** Insertion **A4-3S** that was found in *Ae. tauschii* (603243), DW (Svevo) and WE accessions (TTD160, A1 and A23) (band size = 553 bp). **e.** Insertion **A4-4/5** that was found in DW (Svevo) and WE accession (Zavitan, TTD32, A16b, J1, T1, T2 and T8) (band size = bp). **f.** SS-PCR of insertion **B6-3** was found downstream to a predicted protein coding gene (TRIDC6BG046620). Full site (653 bp) was found in one accession of TA (129526) and almost all accessions examined of WE.


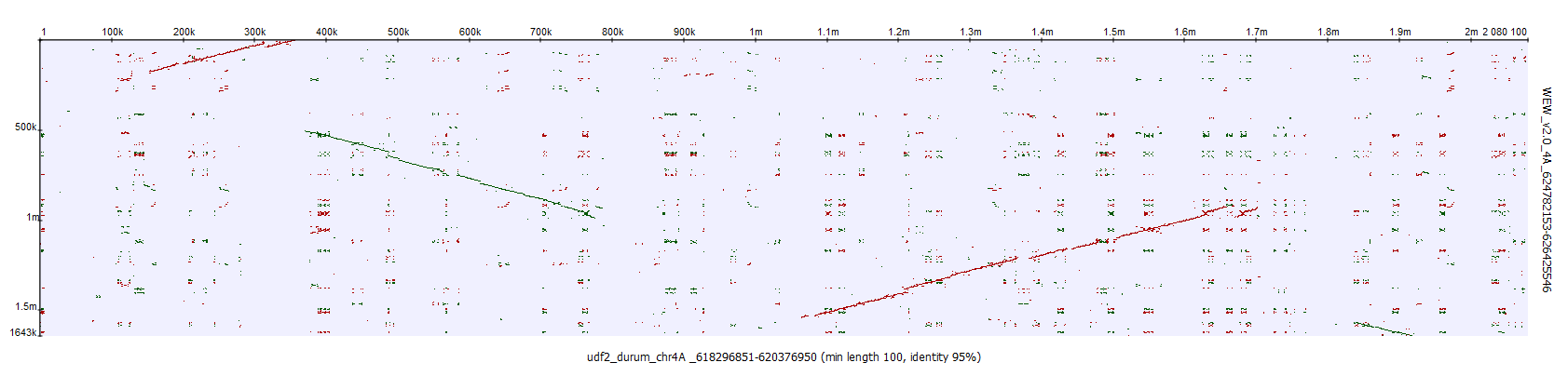


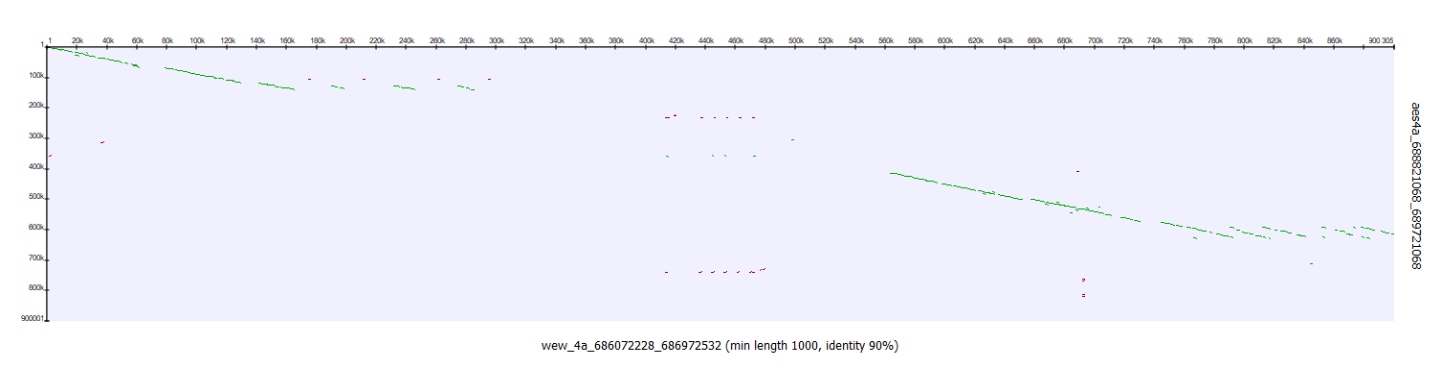


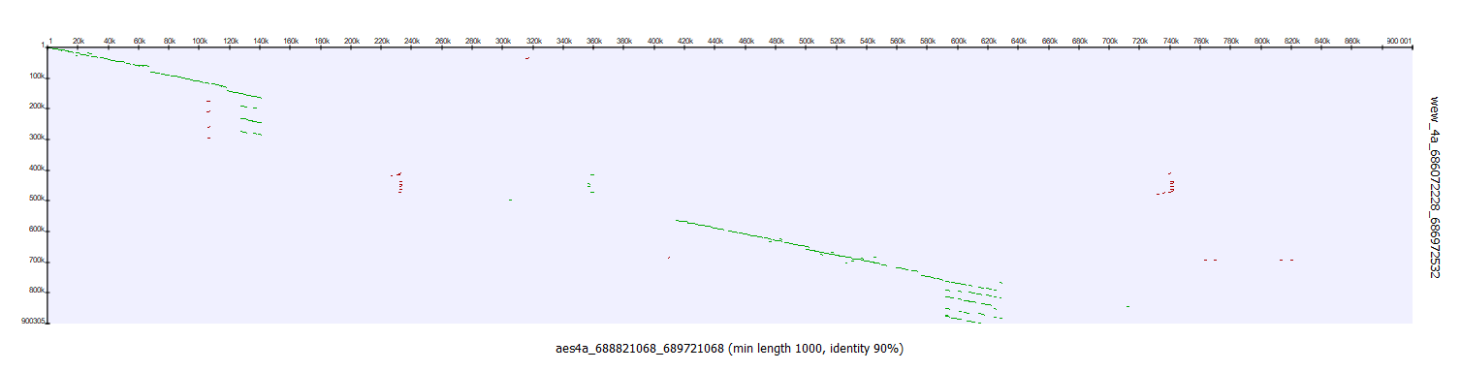


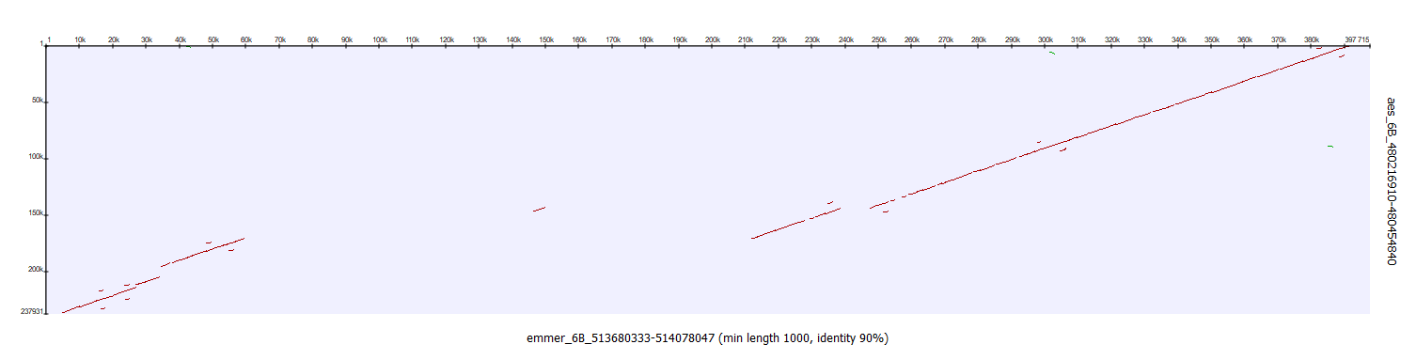


**Supplementary Fig 5. Dot plots of rearrangement cases that involved *Mariam*.
a).** A comparison of the region in DW (Svevo) chr4A that contains an insertion of ~300 Kbp (with *Mariam*) that was not found in TA and WE (Zavitan) and the syntenic region in chr4A of Zavitan.
**b)**. A comparison of the region in WE chr4A (Zavitan) and the syntenic region in TA (bread wheat, Chinese Spring) in which a deletion of ~440 Kbp (including *Mariam*) and insertion of another sequence sized 314 Kbp (in TA) has been occurred.
**c).** A comparison of the region in WE chr6B (Zavitan) and the syntenic region in TA (Chinese Spring) in which a deletion/insertion of ~150 Kbp (including *Mariam*) has been occurred.
